# End-of-life experiences in dementia with Lewy bodies: Qualitative interviews with former caregivers

**DOI:** 10.1371/journal.pone.0217039

**Published:** 2019-05-30

**Authors:** Melissa J. Armstrong, Slande Alliance, Angela Taylor, Pamela Corsentino, James E. Galvin

**Affiliations:** 1 Department of Neurology, University of Florida College of Medicine, Gainesville, Florida, United States of America; 2 McKnight Brain Institute, University of Florida, Gainesville, Florida, United States of America; 3 Lewy Body Dementia Association, Lilburn, Georgia, United States of America; 4 Comprehensive Center for Brain Health, Charles E. Schmidt College of Medicine, Florida Atlantic University, Boca Raton, Florida, United States of America; Foundation IRCCS Neurological Institute C. Besta, ITALY

## Abstract

**Background:**

Dementia caregivers describe knowing what to expect as an unmet need and many are unaware that dementia can be a terminal condition. Dementia with Lewy bodies (DLB) is a common neurodegenerative dementia with unique features which may affect the end of life (EOL). Given the paucity of data on EOL experiences in dementia and unique aspects of DLB affecting EOL, we investigated EOL experiences as reported by caregivers of individuals with DLB.

**Method:**

We conducted telephone interviews with caregivers and family members of individuals who died with DLB in the last 5 years using a semi-structured questionnaire to identify and describe EOL experiences. We used a qualitative descriptive approach to analyze interview transcripts and identify common themes.

**Results:**

Thirty individuals participated in interviews. Key themes included lack of knowledge regarding what to expect, end-of-life time course (including end-of-life symptoms, declines after hospitalization and falls, and varied EOL trajectories), advance care planning, lack of family understanding, hospice, views regarding right-to-die, medications at the end of life, approaching end of life, the death experience, and activities that enhanced end of life. Lack of communication between health care teams and families and difficulty predicting death timing were two frequently expressed challenges.

**Conclusions:**

Study results emphasize the need for improved EOL counseling in DLB, recognition of EOL symptoms, earlier hospice involvement, tailoring EOL care to DLB-specific needs, and clinician-family communication. Suggestions for patient and family education are provided. Further research should confirm predictors of approaching EOL in DLB, identify strategies to improve physician recognition of EOL, and develop tools to aid communication and quality EOL care.

## Introduction

Dementia with Lewy bodies (DLB) is an Alzheimer’s disease-related dementia and one disease under the Lewy body dementia umbrella. Lewy body dementia is the second-most-common neurodegenerative dementia in the United States (U.S.) [1], affecting approximately 1.4 million Americans [2]. Despite this, little is known regarding end-of-life (EOL) experiences of individuals with DLB and their families. Individuals with DLB survive a median of 3–4 years after presentation [3–5] reflecting shorter survival than those with Alzheimer disease (AD) dementia [4, 6] and Parkinson’s disease (PD) [7, 8]. Individuals with DLB have a unique symptom profile which may affect EOL experiences, including hallucinations, paranoia, cognitive fluctuations, parkinsonism, and antipsychotic hypersensitivity [9]. Causes of death in PD are similar to those in non-PD cohorts, particularly for mild to moderate PD [10], whereas individuals with Lewy body dementia commonly die from dementia-related complications and have more than double the likelihood of respiratory death as those with AD dementia [11]. Failure to thrive is the most common cause of death in DLB (65%), followed by pneumonia/swallowing difficulties (23%) [5].

Dementia quality measures recommend “comprehensive counseling regarding ongoing palliation and symptom management and EOL decisions within 2 years of initial diagnosis or assumption of care” [12], but only 22% of families of individuals who died with DLB reported a helpful conversation with physicians regarding what to expect at the EOL [5]. Caregivers of individuals with Lewy body dementia [13], dementia in general [14], and dementia at the EOL [15] and individuals with young-onset dementia and their caregivers [16] also describe knowing what to expect as an unmet need. Awareness of dementia as terminal condition was associated with improved patient comfort at the EOL, but over a quarter of families were unaware that dementia could lead to death [17].

Given the paucity of data on EOL experiences in dementia and unique aspects of DLB impacting EOL, we aimed to investigate caregiver-reported EOL experiences of individuals with DLB and their families.

## Methods

### Approach

Interviews queried EOL experiences of individuals with DLB and their families as reported by caregivers and other family members. A qualitative descriptive approach [18] was used to collect and analyze data. This technique describes straightforward accounts of participants’ views and provides a comprehensive summary without intending to generate or test theory. Consolidated criteria for qualitative research guided study reporting (S1 Checklist) [19].

### Population and recruitment

Participants were recruited through a survey investigating cause of death and EOL experiences in DLB. Survey methods were reported previously [5]. Survey participants were recruited through the Lewy Body Dementia Association (LBDA) and were eligible if they were a caregiver, family member, or friend to an individual who died with a diagnosis of DLB in the past 5 years and able to complete an online English-language survey. At the conclusion of the anonymous survey, respondents were asked, “Are you willing to participate in a brief (<30 min) telephone interview on this topic?” Respondents indicating “yes” received the PI’s contact information. Individuals contacting the PI received an IRB-approved email response with the informed consent form and semi-structured interview guide. Potential subjects were invited largely in order of volunteering but with purposive sampling to achieve representation of different roles (spouses, children) and genders. For example, given that 89% of survey participants were women [5] and interview volunteers were largely women, men were invited to participate in the interviews regardless of email timing to purposefully capture their views. Similarly, after 8 of the first 10 interviews were with a younger generation (offspring, niece) than that of the person with DLB, subsequent invitations preferentially targeted spouses, though additional offspring were also enrolled.

The University of Florida institutional review board provided study approval (IRB201701657). The study was conducted with a waiver of documentation of informed consent. Participants reviewed the informed consent form in advance. Telephone interviews started with an opportunity for questions. After participants provided verbal consent, recording was initiated and the interview started.

### Data collection and analysis

The semi-structured interview guide was developed by the PI (MJA) and revised in response to suggestions from LBDA staff, two experts guiding the LBDA, and three caregivers for individuals who died from DLB. The guide included 11 open-ended questions regarding EOL experiences of the participant and his or her loved one with DLB (S1 File). The PI, a physician specializing in DLB, conducted all the interviews. She had no prior relationship with participants. A professional service transcribed interviews verbatim, therefore member checking was not employed. Participants had the opportunity to receive study results when available.

Analysis was performed using a qualitative descriptive approach to identify, define, and organize themes [18]. Investigators chose this qualitative technique because it describes and summarizes participants’ experiences, consistent with the aim of the study to investigate caregiver-reported EOL experiences of individuals with DLB and their families. Researchers used Microsoft Word 2016 tables to organize data. One investigator (SA), a research assistant with qualitative research experience but without specific topic expertise, independently analyzed the content of 5 interviews to create a draft log of codes reflecting emerging themes and sample quotes (open coding). A second investigator (MJA)–a DLB specialist with qualitative research experience–reviewed and revised the draft coding. These two investigators achieved consensus on emerging themes. SA then analyzed remaining transcripts using a constant comparative technique to identify all instances of the coding framework and items not corresponding to themes in the framework and expand or merge thematic codes (axial coding). MJA reviewed and revised coding again after 20 interviews and after all 30 interviews to further refine emerging themes. Both coders identified subthemes and achieved consensus on themes and subthemes. MJA assessed saturation while conducting the interviews and both coders reassessed saturation following 30 interviews. Additional investigators provided feedback following the initial analysis.

## Results

The LBDA posted the website survey link on 9/1/2017. Over 400 individuals completed the survey in the first week and over 60 volunteered for interviews, so investigators removed the question querying interview interest. Investigators contacted 36 volunteers. Of these, 30 individuals completed interviews, four did not respond after study information was provided, one opted not to participate given scheduling difficulty, and one scheduled an interview but did not attend. Investigators notified 47 additional volunteers that recruitment closed. Interviews occurred between 9/15/2017 and 10/30/2017. Most participants were women (27/30): 13 daughters, 11 wives, 1 sister, 1 daughter-in-law, and 1 niece. Two husbands and 1 son participated. No additional demographic information was collected. These demographics were similar to the survey population, where 89% of the final cohort were women (587/657), 42% were spouses, and 53% were children [5]. Average interview duration was 31 minutes.

### Lack of knowledge regarding what to expect

Lack of information about DLB throughout disease duration affected EOL experiences.

*When we first got the diagnosis of DLB*, *it was kind of like here’s your diagnosis*. *And at that point*, *we were kind of on our own to figure out what that meant*. (#10, son)

Multiple participants reported that physicians never discussed that DLB can be terminal.

*Where I figured out that she was gonna die is from reading all the material I could get*. *But the doctor*, *I don’t think*, *ever said she is gonna die*. *And I think that’s important for this person to know… I think the doctor needs to be very specific with the caretaker*. *Now*, *the patient may not wanna hear it*. (#6, husband)

[When answering what could be improved] *Probably at least addressing and saying that*, *you know*, *this is terminal*, *and these are the types of things that we have to talk about or think about*. *And that never happened… I think that if that had happened*, *maybe we would have been able to get hospice sooner*. (#3, daughter)

Participants commonly self-educated about DLB and EOL by accessing internet resources including the LBDA, hospice sites, and Facebook caregiver groups. Many caregivers read books about dementia caregiving and hospice print materials.

### End-of-life time course

Families described new or worsened symptoms in the last weeks and months of life (Table 1). The EOL period represented decline over 2 months to over a year for many individuals (S1 Appendix), but other participants described that EOL was sudden and unexpected.

**Table 1:** Reported symptoms in the end-of-life period.

Symptom	Exemplar Quotes
Change in appetite	The most significant in the past two months prior was really a change in appetite and she would vacillate between not eating well to eating like she hadn’t eaten, you know, in days. (#7, niece)
Eating Less	He stopped really wanting to eat and it lead to, you know, him being fed. And I ended up feeding him all three of his meals every day, and encouraged him and little by little still he wouldn't eat them. (#9, daughter) He began to, you know, eat less and less… (#10, son)
Loss of ability to swallow	She finally completely lost the ability to swallow. So, obviously at that point, then, she couldn’t—she wasn’t takin’ in any food or water. (#16, daughter) He couldn’t swallow pills, which was never something he could do well to begin with but now he could not do it, and he couldn’t do the liquid because of the aspiration… (#27, wife)
Weight loss	He was dropping weight significantly (#23, daughter)
Weakness	There was a gradual decline in her physical strength. (#16, daughter)
Increased stiffness	Well, I knew he was not doing well because- he started getting very stiff, more than usual… (#29, daughter)
Increased difficulty walking	Now he was walking only with a walker. Before, he was walking with a cane, and I would help him around… (#23, daughter)
Falls	She was gettin’ weaker and weaker, and she was havin’ all these falls…(#16, daughter)
Not getting out of bed	He wasn’t getting out of bed. He was sleeping a lot, and he didn’t wanna eat or drink. (#29, daughter)
Worsening cognition	But end of life… with my mom, was probably about the last month when she really started cognitively declining severely (#8, daughter)
Decreased communication	And at the very end, he was… only talking maybe three or four periods out of a day. You know, he- he—the rest of the time it was just unintelligible noises. (#9, daughter)
Increased hallucinations	I think the biggest thing was, for us was the rapid decline. I mean it was just so rapid. I mean it was just May my dad was at my daughter’s school and then like he started having these hallucinations. He was seeing people. He was seeing like, animals and like stuff, but then it started getting a lot—a bit more severe to where he was actually recognizing the people he saw—like people that he didn’t really see and weren’t really here, you know what I mean? (#19, daughter)
More sleepiness	About three weeks before he passed—and he was getting a little more—you know, he was getting sleepier, logier, more confused… (#22, wife) He was sleeping a lot… (#29, daughter)

*We really had no clue it was gonna come on that fast*. *None of the doctors even*. (#3, daughter)

*The doctors*, *both of ‘em*, *would tell me that they’re shocked at how fast she went down*. (#6, husband)

Six of 30 participants described that EOL occurred after a procedure or hospitalization unrelated to DLB.

*He did fine with the [carotid artery stent] procedure*, *and then the next morning when we went to bring him home*, *he was not able to walk anymore*. *They did a full neuro workup before he was actually discharged later that day… He ended up falling that evening at home*. *We brought him in the next morning to the hospital*, *where he was admitted for presumed pneumonia*, *and he was in the hospital for two weeks and died*. (#1, daughter)

*It was a surgery that was unexpected and everything went downhill from there*, *so that was a very narrow period*. (#8, daughter)

Three participants described worsening after a fall:

*We didn't realize it was the end of his life*. *He was actually going downhill*, *but we had no idea*. *He had a fall*. *He went into the hospital*, *and then he went into hospice*, *and then he passed away… Up until that moment he was at home*, *and I was taking care of him*. *And we had no idea he was that close to*, *you know*, *dying*. (#4, daughter)

A challenge described by multiple participants was “false alarms” about approaching EOL:

*My mother had many near end-of-life experiences in her last nine months of life… she had many times where the nursing home residents or staff would say*, *you know*, *it looked like typically where someone’s nearing end of life*, *and then she rebounded… She went three days once without being responsive and then rebounded and was pretty much back where she was before those three days*. (#2, daughter)

*We were told… three times within the year that she was probably coming to the last few weeks of her life*. *So we were saying*, *estimating about a month*. *And then she rallied*. (#20, daughter-in-law)

### Advance care planning

Individuals with DLB had different wishes for EOL:

*We wanted to keep her at home*. *And we truly wanted to take her out of her home in a pine box like she always wanted*. *And you know what*? *I did that*. (#13, daughter)

*He had talked about this when he was still cognitively aware*, *that he wanted to go someplace… When he would have these clear moments*, *he would say*, *"I want you to put me someplace where I'll be safe and you don't have to worry about me*.*"* (#18, wife)

Families described benefits of advance care planning (ACP) for healthcare decisions and details such as the funeral.

*When he was hospitalized*, *he didn't want tube feeding*. *We had all of that in writing*, *so that was not an issue*, *luckily*. *I'd hate to be the one who had to—and I had POA* [power of attorney], *you know*, *medical POA*. (#15, wife)

*Something else that happened that was exceptionally helpful*: *my parents*, *in a moment of foresight years and years ago*, *got all of their documents in order*, *so I have POA*. *I have advanced directive*, *living—the trust*, *all of that stuff… I have copies*. *All the institutions that needed them*, *the hospital*, *the doctors*, *et cetera*, *the banks*, *they all had copies… Doing as much documentation as soon as you know your loved one has dementia is absolutely critical*. *The more admin prep* [administrative preparation] *you can do ahead of time*, *the easier end of life will be*. *I cannot stress that enough… That I didn’t have to do any of that in my crisis situation was a huge benefit*. (#23, daughter)

*We had our—all of our deaths and burial and so forth*, *stuff all taken care of a few years back*, *so I didn’t have to deal with that*. (#27, wife)

ACP allowed the person with DLB to specify desires.

*He was always very clear about what he didn't want*. *You know*, *he didn't wanna ever be put on a ventilator*. *He didn't want a feeding tube*. *So that helped*, *you know*, *to make our decisions a lot easier because we knew exactly what he wanted and what he did not want*. (#1, daughter)

Family members expressed regret when ACP was not performed.

*I really wish that we would’ve talked more about it before he was too hard to talk to*. (#11, sister)

When individuals pre-specified their wishes, family members advocated respecting those wishes.

*[Name] didn't want any further test*. *They did an EEG*. *They wanted to do a MRI*, *and I absolutely didn't want them to do that*. (#4, daughter)

### Lack of family understanding

Multiple participants described challenges arising from family member lack of understanding. Physician validation of accurate assessments was particularly important in these circumstances.

*We had weekly conference calls with my mother*, *my sister*, *and I*. *I would go down to the unit and sit with the doctor and the social worker and then we would all be on a call together*. *And he would describe what was going on and- and my mother said*, *"Well*, *[name] is explaining this*, *but I think she's just being dramatic*.*" And the doctor said*, *"No*, *she's being really accurate of what's taking place now*.*"* (#9, daughter)

*In hindsight*, *I really wish that*, *at the very beginning*, *at the get-go of when things started to unravel and go down the steep decline*, *when we started hospice*, *I wish that we had done a conference call…*.*had the hospice doctor explain to all of my siblings… what was going on with her*, *where she was at*, *what to expect*, *timeframe*, *that kinda* [kind of] *thing* … *Instead*, *you know*, *I get the information and it’s—as a caregiver*, *you’re already extremely like fatigued and it’s hard*, *hard to remember all that*. *And it’s just overwhelming*. *And so*, *it would have been better*, *I think*, *had we done that*. *I had a sibling that was in denial that my mom was gonna* [going to] *die*. *You know*, *it would have—that would have just helped to… say yes*, *this is true*. (#8, daughter)

### Hospice

Participants described not understanding what hospice is or how it could help.

*This is something kind of a key point… people don’t really know what hospice is*. *And they don’t know that it’s not necessarily—it doesn’t necessarily mean she’s gonna die in three weeks*. (#14, daughter)

*I wish we had done [hospice] much before*. *It would’ve been so much more helpful*. *But I didn’t know*. *Nobody gave us full information*. *I knew hospice was not for just*, *you know*, *in their last days or weeks… but I didn’t understand the scope of what they could do for us*. (#28, wife)

Participants reported that physicians were unaware of hospice as an option or how to arrange it.

*We did inquire about hospice*. *None of our doctors recommended it*. *In fact*, *I called the neurologist*, *and he thought it was too early… He was really just really goin’ downhill*, *and I thought—wow*, *it doesn’t seem too early to me*. (#27, wife)

*I had called both of her doctors… and talked to her family doctor*, *and*, *you know*, *none of them would make a recommendation for hospice*. *They said*, *"Oh*, *you have to contact hospice*.*" Hospice says*, *"No*. *Your doctor has to write an order*,*" until it got to the end*. (#3, daughter)

Many participants felt that hospice was discussed too late. A couple expressed fears that hospice would hasten death. One participant was frustrated by the loss of services associated with hospice:

*I really fought not to put him on hospice because I felt like he should have as much therapy and PT* [physical therapy] *and—- good experiences as possible and not just let him have end of life experience before he was ready*. *That’s the thing with hospice is I think that with this disease*, *dementia*, *the person should be allowed to go on hospice but have the therapy and the exercise to keep their brain going as much as they can*. (#18, wife)

Reported hospice benefits included excellent care for the person with DLB and families, extra support for the patient, education regarding what to expect, avoidance of hospitalization, having a chaplain to help families process, financial/resource support, music therapy, and support through the grieving process. Negative hospice experiences included lack of a timely response to needs, staff lacking compassion, and differences between family and staff regarding medication use (S1 Appendix). One participant was uncomfortable hospice’s religious aspect given her father’s agnosticism.

### Views regarding right-to-die

Multiple participants stressed the importance of dying with dignity and respect. Two participants specifically raised the topic of euthanasia.

*She was in so much pain*, *she asked my sister*, *you know*, *the oldest one*. *One day*, *she asked her*, *“Can’t you just take me out back and shoot me*?*””*, *the next night*, *she said*, *“Can’t you just go in the kitchen and get a knife and stab me*?*”… We should treat humans as least as well as we treat our animals… I mean it’s hard to kill someone*. *I researched*, *you know*, *every way I could think of to help her die… what if I gave her a bunch of Adderall*, *or what if I gave her*, *you know*, *a bunch of*, *you know*, *Xanax*, *or what if I—just*, *it feels—you feel so desperate…* (#2, daughter)

*He would say*, *“Can you call Dr*. *Kevorkian*?*” and that was pretty horrifying to the staff at the nursing home*. *And even after he completely lost his memory*, *he’d have them call and say*, *“Call that doctor for me*.*”… He was*, *and I’ve become*, *a pretty strong advocate of death with dignity… We treat our pets better*. *And I know that’s a very touchy euthanasia issue*, *but he felt strongly about it with good reason*. (#5, wife)

### Medications at the end of life

Many participants described stopping DLB medications (e.g. cognitive enhancing medications, levodopa) near EOL. Occasionally this was associated with a decline:

*I would swear in a court of law that the galantamine worked for her… And when I couldn’t get her to take it… is really when I started noticing the decline*. (#7, niece)

Hospice commonly provided morphine for pain, breathing, or overall comfort. Many participants felt that morphine resulted in a peaceful death, but several participants described morphine as insufficient to address pain or causing paradoxical symptoms. Often hospice needed to supplement morphine with benzodiazepines. Multiple participants described using haloperidol from hospice comfort packs. In one case, this resulted in a severe reaction:

*He looked at the strings hanging down from the overhead lights and he thought they were a noose*. *I mean*, *it wasn't anything you know*, *anything I was worried about*. *I was just relating to [the nurse] how things had been going*. *And she suggested Haldol*. *And I didn't research it… About two*, *two and a half hours since he had his Haldol… He was suddenly sitting upright… Every muscle in his body was clenched*. *His mouth was clenched*. *It was opening and closing*, *opening and closing*. *His tongue was thrusting out*. *He almost bit his tongue off at one point and he was groaning and moaning*, *and he was in terrible pain… His temperature skyrocketed… From there*, *his kidneys shut down*, *and he was gone by Tuesday morning*. (#17, wife)

### Approaching end of life

Participants related that individuals with DLB were often ready for death.

*She had been saying*, *for a while*, *that she just wanted to go to heaven*. *(#3, daughter)*

*She knew it was coming*, *the end*, *and then she started shutting down*. *And she decided not to want to eat anymore*. (#6, husband)

Several participants were frustrated when acceptance of death was followed by a delay.

*When it got to that point*, *there was underlying fear that it wouldn’t be it yet*, *and it was a fear because she was really ready to die finally*. (#2, daughter)

Many family members could tell when EOL was approaching. Experience of the final days to weeks of life, though, varied substantially. Several participants described a difficult prolonged deathwatch, while others described a peaceful process (Table 2). There was widespread agreement that timing of death was difficult to predict and often longer than was anticipated by family members.

**Table 2:** Experiences in the final days to weeks of life.

Theme	Exemplar Quotes
Prolonged death watch	It was just really painful, and she was ready to go, it seemed, although she still wouldn’t die. I mean I really learned a lot about how hard it is to die… It was three weeks where we basically sat deathwatch on her. And for five—the last five days, she didn’t eat or drink anything. Yes, so excruciating. (#2, daughter) I didn’t realize how long that would take. And it was—it was awful… they told me it would be about 10 to 14 days, but it ended up being more like 3 months. (#5, wife) A couple nights I stayed over with her, and one night they called every couple hours and—to like, “Maybe you should come back.” So I went back there, and they said she’s kind of—really labored breathing… And I’m like, is this it? Is this it? Is this it? And then she’d go [gasps], and then we had another, you know, 30 to 45 seconds till she did it—again. It’s crazy. … And then you-you hear these things like, is it that she’s waiting for me to go away so she doesn’t see it, or, you know, what does Mom want? Right?. Maybe this would be the opportunity that I’m not here; she would go. So I stayed away for a couple days… I’m not sure if it was the best thing to do or the wrong thi—I don’t know, but I did, because I needed to rest, and I needed to breathe. Maybe this would be the opportunity that I’m not here; she would go. So I stayed away for a couple days,. I took a couple days off, and she was still with us. (#14, daughter)
Difficult to predict moment of death	She stopped eating and I guess over, um, a period of about six or seven days she held on. Didn’t eat, didn’t drink and slowly, you know, went down. And then did finally, it took a good two or three days where hospice kept saying, “It’s gonna be any minute, any,” you know. She held on. (#13, daughter) And so it’s, it’s very difficult knowing is this the night, is this the day, and you keep asking hospice and they, they, you know, you know, there are certain signs, but no one knows. (#21, wife) Every night, I would think, “Maybe he’ll die tonight.” And the next night, “Maybe he’ll die tonight.” And then next night, maybe, so when it actually happened, you know, I didn’t really know when it was gonna happen. So the hospice people called me, and they said, “His breathing has changed.” And it was about a half an hour away, but by the time we got there, he had already died. (#25, wife)
Peaceful end of life	During those five days, they kept him, you know, very sedated. And he was very comfortable. He only opened his eyes one time during that time and it was really literally hours before he died. [Those days] were peaceful for us. (#9, daughter) It was a good, peaceful ending for him with everybody around. (#18, wife) So that all went very well. I think the very end-of-life experience was gentle and as good as it can be. And I think for [name], they were able to do a reasonable job of managing her worst symptoms. We had established care priorities of minimizing physical and emotional distress. And that meant sometimes that she was medicated to the point where she was sleeping more than maybe was ideal. But if she was awake and crying all the time, that wasn’t a good alternative. (#20, daughter-in-law)

### Death experience

Hospice and nursing staff often alerted families to physical changes signaling nearing EOL, though exact timing was challenging to predict even for medical professionals. Participants who were at the bedside at the time of death described changes including shaking, dark urine, skin mottling, shallow and irregular breathing, and gasping for air. Several participants described that the individual stopped breathing for a minute and the family thought that was the end, but then the individual started breathing again. Participants were distressed when they didn’t know what the final hours and minutes would be like (S1 Appendix).

### Activities that enhanced end of life

Participants described preparing enjoyable outings prior to the final days of life, such as using a wheelchair to spend time outdoors. Numerous participants described value from visiting hospice musicians. Family members played favorite music recordings or television shows. Friends and volunteers read books aloud or recited poetry. Massages and pools helped some individuals. One family printed large photos and placed them around the room. Several participants described the value of simply holding their loved one’s hand and talking to them (S1 Appendix).

## Discussion

Family members of individuals who died with DLB described a lack of information about DLB and the dying process. Death with DLB could be sudden or a gradual process over many months, sometimes with “false alarms.” Various symptoms worsened preceding death (Table 1). ACP improved EOL experiences. Several participants reported difficulties accessing hospice or not understanding its benefits. Hospice experiences were usually but not universally positive. Morphine and benzodiazepines commonly helped, but some individuals had negative reactions. Several individuals received antipsychotics, with a severe reaction occurring in one case. Individuals with DLB were often ready for death by the end. Families described challenges relating to predicting death timing, a prolonged “deathwatch,” and not realizing that it could take weeks for an individual to die. Death was peaceful for most participants.

The emphasis on lack of knowledge is consistent with existing literature. Only 22% of family members reported a helpful conversation with physicians regarding what to expect at the EOL in DLB and most felt unprepared for what to expect [5]. Knowing what to expect is an unmet need described by caregivers in various dementias [13–16]. Many families are unaware that dementia can be terminal [15, 17, 20].

Recognizing EOL in individuals with dementia requires technical skills, systematic approaches, observation, and knowledge for interpreting signs of deterioration [21]. This can be challenging for physicians without DLB experience and most individuals with DLB receive dementia care from primary care physicians [22]. Advanced dementia features include profound cognitive deficits, minimal verbal abilities, loss of independent ambulation, inability to perform activities of daily living (ADLs), and urinary and fecal incontinence [23]. U.S. Medicare hospice benefit guidelines for estimating ≤6 month survival in dementia require (1) an inability to dress, bathe, or toilet without assistance, urine or bowel incontinence, limited speech output, and loss of independent ambulation, and (2) aspiration pneumonia, pyelonephritis, septicemia, multiple stage 3–4 pressure ulcers, recurring fevers despite antibiotics, or insufficient intake to sustain life in the past year [24]. The ADEPT score for predicting 6-month mortality in dementia uses risk factors including age, gender, shortness of breath, pressure ulcers, inability to perform ADLs, bed-bound status, insufficient oral intake, recent weight loss, body mass index, and congestive heart failure to guide prediction [25]. Interview participants described many of these features in the months approaching EOL. Individuals with DLB may also have DLB-specific symptoms such as worsened hallucinations, cognitive fluctuations, parkinsonism (Table 1) and antipsychotic hypersensitivity. Antipsychotic hypersensitivity is a unique DLB feature [26, 27] that is critical to incorporate into hospice planning to avoid reactions that may worsen EOL experiences.

ACP is part of dementia quality measures [12]. Proactive ACP allows active patient involvement and is an important part of dementia care [28]. ACP also improved the quality of dying of nursing home residents with dementia [29]. This is consistent with current results, where ACP improved patient and caregiver quality of life and families without ACP expressed regret.

Two participants raised issues surrounding euthanasia in dementia. This is a complex ethical issue [30] outside the scope of the current study, but emphasizes the need for palliative care targeting quality of life and dignity and hospice providing comfort, support, and symptom control. Despite a clear role for palliative care throughout DLB and hospice care at the EOL, many participants voiced lack of knowledge regarding hospice and lack of education and assistance from their physicians. This may reflect a more general lack of public and caregiver knowledge regarding palliative care [31]. Lack of family and physician knowledge are known barriers to hospice care in dementia [20] and caregivers report difficulty accessing hospice for family members with dementia [32]. Hospice is commonly initiated only in the last weeks of life in DLB [5] and other dementias [32, 33]. Most dementia caregivers describe positive hospice interactions, but some were disappointed by lacking guidance, responsiveness, or individual interactions [32], similar to current findings.

Almost 25% of caregivers for individuals with DLB rate that health state as equal to or worse than death [34]. Death is often viewed as a relief by patients and caregivers [32]. Most individuals with dementia receiving EOL care in long-term care facilities hospice die peacefully [35], but current participants reported distress relating to the duration and unpredictability of the dying process and lack of communication that this was to be expected. Prior research also shows poor communication between physicians and caregivers at the EOL [36, 37].

Throughout this study and prior research, caregivers focused on a need for education regarding DLB, what to expect with DLB progression, and what to expect at the EOL. Such education needs to start with outpatient physicians and continue with skilled nursing home and hospice teams and cover a variety of EOL topics (Table 3).

**Table 3:** Counseling and education topics regarding end of life in dementia with Lewy Bodes.

Topic	Health Care Professionals Most Likely to Provide Education	Considerations for Counseling/ Education
Expected disease duration	PCPs, geriatricians, neurologists, specialists	- Varies widely (<1 year to >10 years) - Median 3–4 years after clinical diagnosis [3–5]
Advance care planning	PCPs, geriatricians, neurologists, specialists, nurses, social workers, skilled nursing facility staff	- Advance care planning is important in early stages after diagnosis [12, 28, 29] - Individuals’ wishes vary - Having plans in place makes end of life easier
Symptoms that may suggest end of life is nearing	PCPs, geriatricians, neurologists, specialists, skilled nursing facility staff	- Loss of appetite, eating less, swallowing difficulties, weight loss - Worsened physical symptoms: weakness, stiffness, trouble walking, falls - Worsened cognitive symptoms: more confusion, increased hallucinations - Decreased communication - Increased sleepiness - Getting out of bed less often
Hospitalization (e.g. relating to surgery, falls) may hasten decline	PCPs, geriatricians, neurologists, specialists, hospitalists, skilled nursing facility staff	- It is common for individuals with DLB to worsen after hospitalization and falls and have a more sudden decline
Experiences over final months	PCPs, geriatricians, neurologists, specialists, hospice professionals, skilled nursing facility staff	- Generally gradual decline - Individuals with DLB can rebound after it appears that death is nearing, leading to “false alarms” of impending death
Role of palliative care, hospice	PCPs, geriatricians, neurologists, specialists, hospice professionals, skilled nursing facility staff	- Palliative care discussions shortly after diagnosis given lack of cure - Early hospice discussions, referrals - Explain role of hospice
Timing of death	PCPs, geriatricians, neurologists, specialists, hospice professionals, skilled nursing facility staff	- Varies widely - Can be weeks after stopping oral intake - Exact details difficult to predict
Medications	PCPs, geriatricians, neurologists, specialists, hospice professionals, skilled nursing facility staff	- Morphine, benzodiazepines common - Individual responses vary - Avoidance of antipsychotics when possible given risks
Ways to promote quality of life at end	PCPs, geriatricians, neurologists, specialists, hospice professionals, nurses, skilled nursing facility staff	- Hospice services (e.g. music therapy, volunteers reading) - Favorite music, television, books - Physical touch - Loved ones’ speaking

PCP: Primary care physicians, DLB: dementia with Lewy bodies

This study is the first to explore in-depth EOL experiences in DLB. Given that many physicians caring for individuals with DLB do not have experience on which to base counseling, identifying common experiences is critical to inform the education needed by patients and caregivers. All participants were U.S.-based except one, potentially limiting generalizability. For example, accessibility of palliative care and hospice will vary by country. Despite sampling to increase male representation, most participants were women, consistent with dementia caregiver statistics in the U.S. [38]. Demographics other than gender and role were not collected. Participants may have volunteered because of particular experiences or strong views, but identified themes were consistent with published dementia research. Recall bias was limited by recruiting only individuals whose loved one died in the prior 5 years.

Study results emphasize a critical need for improved counseling regarding what to expect after DLB diagnosis and through EOL, recognition of EOL symptoms, earlier hospice involvement, tailoring EOL care to DLB-specific needs, and physician-family communication. Research should confirm predictors of approaching EOL in DLB, identify strategies to improve physician recognition of EOL, and develop tools to aid communication and quality EOL care.

## Supporting information

S1 Checklist COREQ checklist. COREQ 32-item checklist outlining the page where each element of qualitative research is reported.
(DOCX)

S1 File Semi-structured interview guide. (DOCX)

S1 Appendix Qualitative coding. Coding tables with additional exemplar quotes.
(DOCX)
