# Supplementary material for: End-of-life experiences in dementia with Lewy bodies: Qualitative interviews with former caregivers
Source: PLoS One. 2019 May 30;14(5):e0217039. doi: 10.1371/journal.pone.0217039 (PMC6542529; doi:10.1371/journal.pone.0217039)
Supplement: S1 File — (DOCX) [file pone.0217039.s002.docx]

**S1 File**. Semi-Structured interview regarding end-of-life experiences in dementia with Lewy bodies (DLB)

**Welcome:** Thank you for participating in our survey about end-of-life experiences with someone with dementia with Lewy bodies, and for your willingness to be interviewed. As we mentioned at the beginning of the survey, no research has looked at the topic of what the end of life is like for people with dementia with Lewy bodies and their family members. This makes it very difficult for doctors to help patients with DLB and their family members know what to expect. We want to learn more about peoples’ experiences with dementia with Lewy bodies at the end of life so that we can help people with dementia with Lewy bodies, their families, and doctors manage this stage of the disease better.

We will be audio recording the discussion so that we review the conversations further later. There are no right or wrong answers, just different points of view. You may choose not to answer any question and we can stop at any time. Do you have any questions?

**Semi-Structured Interview:**

1. Tell me about you and your loved one’s experience when he/she was near the end of life.

2. What went well during that time?

3. What could have made that time of life easier or better?

4. What decisions had to be made before your loved one died?

(Possible prompts: For example, what decisions needed to be made about whether or you or your loved one could remain at home, or whether you did or did not want to work with hospice?)

5. What kind of discussions, if any, did you have about autopsy or brain donation?

6a. Who on your healthcare team (if anyone) mentioned the option of hospice to you?

6b. (If hospice discussed) When was hospice mentioned to you as an option?

7. What were your thoughts about hospice care, both at the beginning and over time?

8a. If you and your loved one used hospice, were you satisfied with when it was started?

8b. In retrospect, what would you have changed about your hospice decisions (or not)?

9. What medications were used at the end of life to provide comfort care?

10. Looking back, are there any things you would do differently about how you approached this time in your and your loved one’s life?

**Ending Question:**

We are coming to the end of the interview. What are the most important things that you would like others to know in order to help improve the end-of-life experience for other people with dementia with Lewy bodies and their families?

**Closing:**

Thank you so much for your willingness to share your thoughts with us. When we have finished all our interviews, we will be developing a summary of what people said. Would like to receive a copy? (If yes, ask for preferred address, either email or postal.)

Thank you again.
