# Supplementary material for: End-of-life experiences in dementia with Lewy bodies: Qualitative interviews with former caregivers
Source: PLoS One. 2019 May 30;14(5):e0217039. doi: 10.1371/journal.pone.0217039 (PMC6542529; doi:10.1371/journal.pone.0217039)
Supplement: S1 Appendix — Coding tables with additional exemplar quotes. (DOCX) [file pone.0217039.s003.docx]

**S1 Appendix. Qualitative Themes, Subthemes, and Exemplary Quotes for “End-of-life experiences in dementia with Lewy bodies: Qualitative interviews with former caregivers”**

| Theme | Quotes (Interview #) |
| --- | --- |
| **Lack of conversation/ discussion with physicians** | **No discussion of fact that dementia can be terminal**  probably at least addressing and saying that, you know, this is terminal, and these- are the types of things that we have to talk about or think about. And-and that never happened. … That never happened. Um, yeah. And-and I think that if-if that had happened, maybe we would have been able to get hospice sooner. (3)  where I—where I’ve figured out that she was gonna die-d-die is from reading all the material I could get. But the doctor, I don’t think, ever said she is gonna die. And I-I think that’s important for this person to know, and you know, you really don’t know when they really got it.. the lifespan of the Lewy body patient is seven to eight years, I believe, and depending on when they’re diagnosed, how long do you have to go? I think the doctor needs to be very specific with the caretaker. Now, the patient may not wanna hear it. That, I think, would be up to the caretaker. My wife would never wanna know she was gonna die. I know that. She felt—I-I-I’m certain she felt she was gonna die, you know, soon . (6)  **No discussion of what to expect at end of life**  none of the doctors even—I'd asked them about hospice, and none of them even recommended hospice. Um, there was really no discussion of what the end of life was gonna be like for her. (3)  no conversations with any doctors about end of life, um, or what that was going to be like, or what to expect, or what to plan for. I mean, nothing, nothing (3)  I'd asked them about hospice, and none of them even recommended hospice. Um, there was really no discussion of what the end of life was gonna be like for her (3)  That was one of the main problems. So now that I've done my research, I realized that he was having failure to thrive, um, and, you know, he should have been on— (15)  Yes and no. Uh, the nurse who ‑ who ran the home was telling me symptoms that she was seeing—that was still only the last week. Um, so some—again, I—I've done my research now, and I understand these symptoms better. So she was saying things like, you know, his cheek—the skin on the cheekbones was tightening, and he was—‑ breathing more, uh, ragged, and he was, you know, sleeping all the time. You know, all of those kinds of signs that—‑ the end was coming. He didn't want to eat at all, even pureed food, so— (15)  *When asked if caregiver felt prepared for what to expect*  I think educate you more on the disease… - for me. For example, when we found out she had Lewy body disease, the original neurologist said she has Lewy body dementia,— but didn’t really explain how bad that disease was (6)  *Speaking of how doctors could have helped more*  I believe that knowledge is really key for people to-to be able to process what’s happening. And so, more information is better. And if, uh, if it’s a tough thing, bluntla—bluntness with compassion is so much kinder than the platitudes and— (8)  **Lack of guidance**  They listed it as just failure to thrive, which was— a term I had never heard of before. So—and just the-the whole thing was—I don't know. I just didn't feel like I was getting any guidance from anybody. (3)  And, you know, both of us were like, "W-w-we don't know what to do," you know? (3)  her distress caused, obviously, you know, the family distress and everybody’s trying to fix it. And, um, so, that was—you know, that caused, uh, confusion on my part, because I would look to the medical community, the hospice, as to how to resolve this distress. And there-there was no great answer. There was—there was no answer. (8)  I think it would’ve been good if somebody—when we first got the diagnosis of DLB, it was um, it was kind of like here’s your diagnosis. And at that point, we were kind of on our own to figure out what that meant um, you know, in terms of the disease itself. And so, really, nobody stayed with us to make sure—there was no follow-up with us to make sure we were okay, and that we understood. (10)  I—and I don’t understand. I-I’m not sure why that—I mean I had no idea until I started, uhm, you know, we started this-this, uh, journey with my dad that things were the way they were, uhm, and especially for somebody with that type of illness… (19)  **Doctors don’t ask what patients want at end of life**  No. No. Not at all. Not at all. (3) *Speaking of doctors asking what patient wanted for end of life*  **General Discussions about end of life**  we-we did talk about it in generalities (4) *Speaking of end of life*  **Family’s Poor communication with provider**  I don't feel that from his PCP perspective, when she would take him in and explain things to the doctor, like that he was declining, that she was—my mother, that is—was offered, um, any type of additional care—- for perhaps like end-stage—the end-stage Lewy body dementia. Like hospice or palliative care support. (1) *Speaking of having to take over caregiving from Mom- who was previously primary caregiver*  It is so hard being a caregiver. And I think it's really unrecognized by people who aren't living through it. Um, and doctors don't do a good job of supporting it a lot of the time. (17) |
| Long road, suffering for years | “he was just constantly frustrated. Um, and for two years, he wasn’t able to, you know, stand or walk or meet any of his needs. He had to be fed, and, um, it was, yeah, just absolutely excruciating. (2)  It was just grueling. It was a long road (5) |
| Time from diagnosis | my dad was diagnosed about seven years ago with dementia with Lewy body (1)  so she was diagnosed, um, three years prior to her passing. (2)  he died about five years after being diagnosed. (4)  It was—well, [sighs] th—we began to see, you know, really, the most significant changes in—over the past year. So, she was diagnosed in two thousand—uh, I-I’ve lost track. I think it was 2010. It may have been early 2011, but I wanna say 2010. (7)  because it was 13 years when he was diagnosed with it, and, um, it was just unbelievable at the end. (13)  my dad had, uhm, Parkinson’s. So, uhm, he-he had it for a while, so he was, uhm, diagnosed with Parkinson’s maybe six years ago. Because of his stage IV cancer, they thought that they’d never get to a point where we would have to, uhm, where we would have to really be concerned with the Parkinson’s. He was really independent. He was doing all of his, you know, daily activities and all of that on his own, very, uhm, active and, uhm, and then probably, uhm, I wanna say like in May he started having very severe hallucinations and then that’s when his neurologist had diagnosed him with Parkinson’s, but with a, uhm, I guess a—I don’t really wanna say a symptom, but I guess that’s kinda what they called it, with the symptom of Lewy body dementia. (19)  when I hear of someone whose parent has had Alzheimer’s for 20 years and hasn’t known them for 20 or so years, um, I—my hear breaks for them. I—I’m—I feel fortunate or blessed, and mother was blessed, that her illness, this illness and the decline was four years. As far as from when we became aware of it. (24)  he was diagnosed with Parkinsonianism - in 2002. And he died in March—uh, the end of March, 2016.  So, this was a journey for 14 years. (26) |
| Slow decline over months (at end) | “we had noticed that he was declining over the past six months” (1)  his decline was uh, I don’t know—I don’t know if it’s considered precipitous or not. It was over a period of about 18 months um, where he uh, fell ill and, you know, actually was—it kind of came from what we believed was a little heart attack, and then an admission into the hospital. (10)  But uh, you know, he sorta just went through, then, a steady decline. (10)  … the most significant in the past m—two months prior was really a change in appetite and, uh, and she would vacillate between not eating well to eating like she hadn’t eaten, you know, in days. Um, so that was—and it always did my heart good to see her eat. (7)  She was under hospice for over a year, uh, and, uh, of course graduating in decline. (12)  So I was the one that brought up Hospice. Um, to our primary doctors. And, you know, oh well she’s not terminal and I’m reluctant to take her off some of the meds, and that sort of thing. Um, my mom ended up being on Hospice for almost a year and a half before she passed. And, um, you know, we, she kept, you know, her, her condition declined, but it declined slowly and— we were able to get a recertified.. (13)  but then about three months before she died, she had, um, what was classed as a brain incident, and-and we never really got to the bottom of exactly what happened. Um, but, um, she had this sort of fit, and-uh, and-and-and after that, she lost, um, her ability to speak and swallow, and, you know, she couldn’t—- she couldn’t walk or anything. Um, and we thought, to be honest, that she wasn’t gonna make it after that incident. (15)  ...me mum was actually doing all right. She—I mean, and obviously there was-there was a-a gradual decline in her physical strength and-uh, and the cognition, just as you would expect. Um, but then about three months before she died, she had, um, what was classed as a brain incident, and-and we never really got to the bottom of exactly what happened. (16)  so it was kinda like a-a tra-traumatic, um, event that then-that-that—and it just went downhill from there, really. Um, but I would say that, you know, she-she was gettin’ weaker and weaker, and she was havin’ all these falls, but the-the-um, the thing that really did—you know, ended it, I think, was that she-she-she finally completely lost the ability to swallow. So, obviously at that point, then, she couldn’t—she-she wasn’t takin’ in any food or water. So, that—you know, and-and once that happens…(16)  Um, I think the beginning of May—I mean he was—he was dropping weight significantly. Now he was walking only with a walker. Before, he was walking with a cane, and I would help him around…. He was in hospice maybe two, three weeks, and he passed. He passed on the—maybe three weeks—he passed on the 6th of June. (23)  The same neurologist, about six months out, right before Christmas, told me that he had—he—it was now time to um, to call hospice. And uh, he told me that uh, he-he-he was—he had done all he could. (24)  at the beginning of this year, 2017 um, it-it became obvious that he was, um, progressing a little more quickly. .. Four to five months (of healing from skin cancer removal)…toward the end of that three-, four-month period, um, she mentioned something about hospice. . .And I said, well, um, I didn’t really think we were ready for that. I mean, he was still up and around. (28) |
| Sudden deterioration at end | with my mom’s, um, disease, hers was more catastrophic at the end. we had to make the decision to stop, uh, the carbidopa-levodopa…. we had to make the decision to stop, uh, the carbidopa-levodopa… steep decline. Her disease was pretty steep, you know, throughout the whole five years of, you know, when we look back on it. But end of life, for me, for—we—you know, in my—in my mind, with my mom, was probably about the last month when she … really started cognitively declining severely. (8)  And it was about the last month of his life where he really, really started to um, um, decline. You know, he had signs of uh, you know, congestive heart failure um, you know, he uh, he began to uh, uh, he began to, you know, eat less and less…. (10)  **End-of-life period was a surprise, sudden**  he was admitted for presumed pneumonia, and he was in the hospital for two weeks and died, um, about—well, it was exactly two weeks later….although we—we had noticed that he was declining over the past six months. Um, we were very shocked at how quickly it actually went downhill from the initial, um, procedure and then the fall and then - him passing. (1)  we really had no clue it was gonna come on that fast. -none of the doctors even (3)  we didn't realize it was the end of his life. Uh, he was actually going downhill, but we had no idea. He had a fall. He went into the hospital, and then he went into hospice, and then he passed away… up-up until the—that moment he was at home, and I was taking care of him. And we had no idea he was that close to, you know, dying (4)  he had his days, where he-he c—he could talk to me, you know, and we would talk. So, um, you know, it's—it-it wa—it was sudden. I-I expected that we would be putting him in a nursing home, and he would be there for a while. So it-it mercifully came to an end rather quickly. (4)  the doctors, both of ‘em, would tell me that they’re shocked at how fast she went down. And then, oh, I would say maybe two weeks before she died, they said, “It’s now time we have to bring in hospice,” and that’s what they did. (6)  …his last stages were very quick. Um, it was a result of him falling again [chuckles] and hitting his head again (9)  it was a- it was a very quick decline. He stopped- well he was able to talk. Um, didn't make a lotta sense some of the time, um, but he stopped really wanting to eat and it lead to, you know, him being fed. And I ended up feeding him all three of his meals every day, um, and encouraged him and little by little still he wouldn't eat them. And at the very end, he was, you know, choking, you know, he had the- total dysphasia. Um, as well as- as only talking maybe three or four periods out of a day. You know, he- he—the rest of the time it was just unintelligible noises. (9)  it was about the last month of his life where he really, really started to um, um, decline. You know, he had signs of uh, you know, congestive heart failure um, you know, he uh, he began to uh, uh, he began to, you know, eat less and less, and so he-he… you know, kinda that was how-how things ended for him. (10)  for us it was—it wasn’t maybe a—you know, as-as painful as it could’ve been. I mean you-you kinda did go through sort of this, you know, wow, quick, it was definitely a progression that you could see, you know, the end was coming. It was going to be over the next few weeks. for us it was—it wasn’t maybe a—you know, as-as painful as it could’ve been. I mean you-you kinda did go through sort of this, you know, wow, quick, it was definitely a progression that you could see, you know, the end was coming. It was going to be over the next few weeks. (10)  {Wife} had taken a—uh, it was quite a change overnight, uh, to her, uh, condition, and the time had probably arrived that we needed, um, consider administration of, uh, morphine…. the doctor still called us, um, that morning to get permission to administer the-the morphine at a minimal-minimum dose. Uh, and, uh, before it was—uh, this is probably another level that she’d reached that the end of life was probably approaching, and, uh, she probably would, uh, pass away within the next several weeks to a month, so we’re near the end. So, again, this is the kind of thing we were expecting to hear, and we went forth with the, uh, administration of the morphine. (12)  ..and of course, we didn't know how end his near was. He was ‑ he was only two weeks in the home before he passed away. He never stood up again. That was one of the main problems. So now that I've done my research, I realized that he was having failure to thrive, um, and, you know, he should have been on—… He suddenly came down with the symptoms of the infection. I thought he was hallucinating. He refused to eat—‑ and said there was concrete all over his food. And I think his addled brain was telling him he shouldn't eat— because there was something going on wrong inside. And ‑ and this was, like, 24 hours of him not eating, and then he got a fever, and I said, oh, we need to go to the ER. So— he had been on a sort of a plateau—‑ with the Lewy body and ‑ and, you know, doing okay. His symptoms were getting worse, but that was expected. He did not rebound. No. Did not at all. No. He was on pureed food and, um, you know, immobilized in bed for the rest of his life (15)  he actually passed away then on that Sunday, but on Monday when I saw him, he told me to have a good trip… You know, just really monotone talking. Um, but he did know what was going on—- so I did not anticipate that it was gonna happen that week—that week. I mean we knew, of course, the trajectory of what was going on but they said to me that even on Thursday he seemed to be—you know, he was wandering around, you know, like he—the facility where he was at, they were able to walk around. It was 15 people in his, um, group—his hallway or neighborhood, as they called them. And they said that there was really nothing different about him. And as I said, then he just didn't wake up on Friday morning. (18)  So I think the biggest thing was, uhm, for us was the rapid decline. I-I mean it was just so rapid. I-I mean it was just May my dad was at my daughter’s school and then like he started having these hallucinations. He was seeing people. He was seeing like, uhm, animals and like stuff, but then it started getting a lot—a bit more severe to where he was actually recognizing the people he-he saw— like, uhm, people that he-he didn’t really see and weren’t really here, you know what I mean? (19)  My dad’s decline was very rapid. He was diagnosed in May and then he-he passed on, uhm, August 27th… And he just—he just went downhill (19)  he fell Wednesday night. He had the surgery Thursday night, Thursday evening. Friday is when we made the decision. They had the hospice come in, the palliative and hospice care (19)  about three weeks before he passed—and he was getting a little more—you know, he-he was getting sleepier, logier, um, more confused, um, and he was pretty much wheelchair-bound by then, but that had only been for, uh, a few weeks, less-you know, less than a month. And, um, one day we went in there. I wanted to visit with my son, and he was—mmm, nurse said she’d fed him lunch. He was extremely tired. She put him to bed, and he never got up, and he was dying for close to three weeks. (22)  Um, overnight—that was Monday—overnight his breathing changed, and by Tuesday morning, um his eyes were half open, and um breathing very heavily; uh, you know, [breathes heavily] something like that. Um, it was later that afternoon that he passed. And hospice-hospice came by every day starting that previous Friday. (23)  it was only about two weeks that we had hospice before he passed on, and, um, what happened was he got aspiration pneumonia. And he—because he couldn’t swallow pills, which was never something he could do well to begin with but now he could not do it, and, um, he couldn’t do the liquid because of the aspiration, they said the only other option was to put him on IV antibiotic… but he went into the hospital on Sunday, late afternoon, and he passed away on Thursday… in the hospital (27)  Well, I knew he-he was not doing well because- he started getting very stiff, um, more than usual. He wasn’t getting out of bed. He was sleeping a lot, and he didn’t wanna eat or drink. And I knew right then. It was very sudden, you know. Just happened real suddenly and uh, he just, um, got really stiff. And I called hospice and got hospice in, and that was a bad situation. (29)  And, um, he was there for about two-two years, maybe, and then, boom, all—that-that—it hit really fast where he got super stiff, boom, you know, he just—told you about the death. (29)  Some guy told me his mother said that, um—he was tellin’ me his mom had it, and then, boom, she just [inaudible 24:04] super fast and died. And, uh, my dad sorta did, too. It kinda- He was a little more tired, but it did kinda happen rather quick, I thought (29)  So it was—the real end came about eight days before he passed away. And that happened—what happened, and at that point we’d had him, he had an atrial stenosis. And they said if it got—when it got where they couldn’t hear it anymore, then-then it was too stiff—it was, you know—it was in the end of it. And so about that time is when that happened. (30)  **Sub theme: After Procedure**  “he did fine with the procedure, and then the next morning when we went to bring him home, he was not able to walk anymore. Um, they did a full neuro workup before he was actually discharged later that day. We took him home. He ended up falling that evening at home. We brought him in the next morning to the hospital, where he was admitted for presumed pneumonia, and he was in the hospital for two weeks and died” (1) *Speaking of carotid artery stent.*  It was, although we—we had noticed that he was declining over the past six months. Um, we were very shocked at how quickly it actually went downhill from the initial, um, procedure and then the fall and then him passing” (1)  put it in-in your mind ..And he certainly got worse after each procedure. And the anesthetic, I think, really screwed him up. I-I don't know if people are aware. I certainly wasn't, 'til I sort of—you know, that, uh, anesthe—when people have surgery and anesthesia, with those conditions, it's horri— it really, I think—deteriorates things further (4)  It was a surgery that was unexpected and everything went downhill from there, so that was a very narrow period. (8)  Uh, he'd been hospitalized with a gallbladder infection, and, um, he was okay mentally, but physically, he wasn't doing well. I had to decide whether he should have surgery and decided not, and so other issues, uh, happened. So after he'd been in the hospital for about two weeks, the doctor said, "We'd like to have a meeting with you." And she had a group of about four people, including a social worker and they started out by saying, "Have you considered what would happen if your husband could not go home from the hospital?" Ka‑boom. No. [Laughter] Uh, and so they gave me 24 hours to find him a place to ‑ to stay. … He was ‑ he was only two weeks in the home before he passed away. (14)  he got sick with this gallbladder issue, took a step down, and was in the hospital, really didn't rebound from that. (15)  he had cataract surgery the next day and then that’s when the doctor had told us with the anesthesia and, you know, all of this, he didn’t know if my dad would ever really recover and he didn’t. they said if he did for somehow come out of it, then, you know, we wouldn’t have to stay in hospice, but we decided—we-we had to make that decision to move my dad to hospice care on Friday and then he passed on Sunday. (19)  He was independent. He was walking. He was eating, showering, changing, all that stuff on his own, and he was up until the time he went into the hospital in July. (19)  **Sub theme: After Fall**  we didn't realize it was the end of his life. Uh, he was actually going downhill, but we had no idea. He had a fall. He went into the hospital, and then he went into hospice, and then he passed away… up-up until the—that moment he was at home, and I was taking care of him. And we had no idea he was that close to, you know, dying (4)  …his last stages were very quick. Um, it was a result of him falling again [chuckles] and hitting his head again (9)  about nine days before she died she had a fall. And she’d had many falls but mostly fell against a piece of furniture or against the bed and then slid to the floor. So she hadn’t had any visible injuries of any of her earlier falls. This time, she fell, was found by an aide on the floor of her apartment with her walker overturned beside her, and she knew she’d hit her head. still able to walk to the bathroom but with spending most of her time when she wasn’t in the bathroom or having a meal, she was in bed and most of that time was asleep. And then about-um, about five or six days before she died, she was u—became unable to walk, to bear weight. (20) |
| Choosing hospice after an acute illness | he developed an acute GI bleed. And then after that, he—it was, like, 48 hours later he developed a really high fever. And when they reimaged his chest, they saw bilateral pneumonia. So at that juncture, we knew he probably didn't have much fight left and we decided that we would just allow him to be made comfortable.” (1)  I spoke to the—the hospice doctor who came in, and—oh, oh, yeah, cuz he was still—yeah, he was still not hospice because he was in the rehab part of the facility. Um, and I said, you know, “I’m thinking that maybe this is the time that we should put him on hospice, at end of life hospice.” (25) *Speaking of hospice after pneumonia acquired in rehab facility*  I spoke to the—the hospice doctor who came in, and—oh, oh, yeah, cuz he was still—yeah, he was still not hospice because he was in the rehab part of the facility. Um, and I said, you know, “I’m thinking that maybe this is the time that we should put him on hospice, at end of life hospice….., given that he had Lewy bodies, he was—he was doing pretty well with that, but, um, when he developed the pneumonia…. So, at that point, what we did is we moved him into the nursing home wing, you know, from the rehab, same facility. Moved him into the nursing home wing, and, um, they started with the hospice there. (25) |
| Swallowing problems contributing to end of life | he had issues with choking and he-he choked at—chocked on food a couple times and had trouble swallowing, and-and, um, they made me aware that that’s where it was leading, and— Um, I-I didn’t actually perceive that, um, not remembering to swallow would be an issue, Um, and I wa—I didn’t really—when they explained to me how awful feeding tubes were, they didn’t really explain to me how awful not eating was. And I clearly understood that we weren’t gonna be able to teach him, retrain him. that this was a new normal. Um, but I didn’t realize how long that would take. And it was—it was awful (5)  And trying to coax her to swallow and to, you know, um, it’s those types of things that caregivers are not adequately, you know, how do we deal with this and what- uh, that type of thing. (13)  Um, but up until that point, she was doin’ really well. Um, so it was kinda like a-a tra-traumatic, um, event that then-that-that—and it just went downhill from there, really. Um, but I would say that, you know, she-she was gettin’ weaker and weaker, and she was havin’ all these falls, but the-the-um, the thing that really did—you know, ended it, I think, was that she-she-she finally completely lost the ability to swallow. (15)  Well, what happens now?” And she said, “Well, nothing ‘cuz, you know, she can’t swallow. She can’t swallow.” And that-that just seems to be almost the end of it. It’s like, we tried. It’s gone down into her lung once, and we’re not really prepared to try anymore.  ….but, um, a-about three—two to three days before she died, she started to get this really gurgling-type sound in her chest, as though—and it sounded as though there was a lot-a lot of liquid and mucus on her lung. So, um, we kinda, like, went to the nurse ‘cuz, I mean, it was—seemed to be not very pleasant for her, and it was pretty dis—if you know, distressing for us as well, to be honest. So they tried to suction it out a couple of times, which was causing distress because obviously that was makin’ her gag. (16) |
| Rallying/rebound  False alarms on death (over weeks-months prior) | my mother, um, had many, um, uh, near end of life experiences in her last nine months of life, where-… she had many times where the nursing home residents—or staff would say, you know, it looked like typically where someone’s nearing end of life, and then she rebounded. (2)  she went three days once without being responsive, um, and then rebounded and was pretty much back where she was before those three days (2)  Because, as I said, she had several, um, uh, what appeared to be end of life experiences, and then she would rebound from them. (2)  And she—my aunt actually kinda rallied a little bit, like I said, you know? And, you know, she kinda ate on Tuesday, ate a great dinner on Wednesday, and had stuffed peppers. She loved stuffed peppers. She ate a very good supper on Wednesday. Thursday, I made [clears throat] spaghetti with her sauce recipe, and she ate very well, and—but then, the weekend came, and she was more back to sleeping, um, mostly. And didn’t really have much-much input, you know, intake. Like I—like I said, I could get an—the Ensure shake… (7)  she was at my house, I would say, another week, I’d have locks on it— because uh, when they start goin’, they-they got a lotta strength in ‘em. They got a lo—a ton of strength. (7)  So it was kind of a little bit of a rollercoaster towards the—towards the—-end, right? So she’d stop eating maybe, or stop drinking. They’d let us know, but then the next day, she would, so it was—it was almost like the moments of lucidity that used to come and go. (14)  It was like she kind of was eating or drinking, and so it was—you were kind of in the clear again. You could breathe again. And then you get to the point where now she’s not again. And then it was finally that she just was fre—just was too weak and-and—to eat or drink at all. (14)  Um, but then towards the end, it was that um uh she started not being able to take as much foo—you know, food in. There-there were many—there were many times where it’s-it’s-it’s starting to happen, and then it kinda wouldn’t. (14)  …she had this sort of fit, and-uh, and-and-and after that, she lost, um, her ability to speak and swallow, and, you know, she couldn’t—- she couldn’t walk or anything. Um, and we thought, to be honest, that she wasn’t gonna make it after that incident. But she did-she did then start to improve, um, and she-she got-she got her speech back, and she got her swallow back. And, um, although she was very unsteady on her feet, she-she did—you know, she could walk, you know, with help. Um, so we were quite hopeful (15)  Um, but then about three months before she died, she had, um, what was classed as a brain incident, and-and we never really got to the bottom of exactly what happened. Um, but, um, she had this sort of fit, and-uh, and-and-and after that, she lost, um, her ability to speak and swallow, and, you know, she couldn’t—But she did-she did then start to improve, um, and she-she got-she got her speech back, and she got her swallow back. And, um, although she was very unsteady on her feet, she-she did—you know, she could walk, you know, with help. Um, so we were quite hopeful…(16)  Well, he's doing very well," um, and they were gonna consider not doing it. They said, "You—we can—you can call us up anytime," but, again, because this disease—for me, what I—my observation is that he would level off, and things would be just level. Whatever level it was— it would be just kind of going along. And then he would—he would make dramatic change, and it would always go down—you know, always downward. Then, of course, he would do that, so hospice stayed with us… (18)  during the course of that year, we had a couple of times when she was really in a trough, and that was—  that was part of why I think—that’s related to why it’s so hard to tell people what to predict at the end of life— cuz we were told twice during that year—well, actually three times within the year that she was probably coming to the last few weeks of her life. So we were saying—mmm, estimating about a month. Um, and then she rallied. She rallied in a big way on her 99th birthday, [laughter] and seemed to sustain that high for quite some time. So that was in January of this year. And then, uh, again in June she was in another trough and then again in August. (20)  mother would have dips, but then she would bounce back. And I was like how do you, you know, how do we proceed with this? And the hospice uh, nurse or social worker said well, you know, the dips, the highs and the lows, the dips and the bounce-backs will become uh, less dramatic. And uh, and she will bounce back, you know, less and less. Or when she bounces back, she’s not back up to par. And then she was right. It just was very gradual and uh, and you saw that the decline, you know, was occurring. (24)  Um, and there was one day—cuz they did continue to bring him into the dining room for about two, two and a half weeks, until he just—he-he was really at that point, uh, became bedridden. There was one day, maybe about a week later—and I was feeding him, um, what I call milkshakes, [laughter] chocolate milkshakes, which was Ensure. Um, and-and he-he would maybe eat a teeny bit and we were trying to puree it. And then there was one day and he just went to town. I mean, I was all excited. “Oh, my gosh!” Uh, you know? And, um, but he reverted back. I-I don’t know. Maybe—they said that he was, um, uh, he was, what do you call it, um, aspirating his, um, food in-into his lungs. (26)  You know, [sighs] I didn’t know how much of it—of what he was experiencing was a dip down because that would happen, and then he’d bounce back up again - um, or if it was that he was moving quickly or if that was normal, or if he was even moving slower because early on, everybody who seemed to have any kinda knowledge said, “Well, he’s-he’s progressing very slowly, and that’s a really good thing.” (28) |
| Pre-expressed wishes from patients, advance directives | he had a DNR, and they took him to the hospital and started pumping him full of antibiotics. I think he—you know, he had already decided—as-as a—as a paralyzed person, he had already decided that, if your quality of life s-stunk, you know, it’s over (5)  And it’s a pink piece of paper, and, uh, different categories of, uh,no tube-feeding and, uh, this type of thing. And it’s signed by, uh, uh, if possible, the patient, which we did, uh, uh, before—um,] did this some time ago. So, all the legal steps—the decisions were made. (12)  I made that promise to my mom and dad. I’m the only girl. I have four brothers. Um, we wanted to keep her at home. And we truly wanted to take her out of her home in a pine box like she always wanted. And you know what? I did that. (13)  Like when he was hospitalized, he didn't want tube feeding Yeah. We had ‑ we had all of that in writing, so that was not an issue, luckily. I—I'd hate to be the one who had to—and I had POA, you know, medical— POA. (15)  We did have a-a—he did have his CPOA in place, and I, um—I kind of pushed for that, uh, in 2015. Um, I pu—I could see that hi-his thinking wasn't as good as it—- should be, and, um, I just wanted to make sure everything was in place. But he seemed to not recognize what the doctors were saying to him, when they even mentioned Lewy bodies, you know? (17)  So now towards the end of his life—sorry—so then he went into the facility, and on one hand he was—he had talked about this when he was still cognitively a—aware, that he wanted to go someplace. He wanted to be put someplace. When he would have these clare moments—clear moments, he would say, "I want you to put me someplace where I'll be safe and you don't have to, uh, worry about me." (18)  something else that happened that—eh that was exceptionally helpful: uh, my parents, in a moment of foresight years and years ago, um got all of their documents in order, so I have POA. I have adva—eh—dire—uh advanced directive, living—uh the um trust, all of that stuff. All of those documents were finalized. I have copies. All the institutions that needed them, the hospital, the doctors, et cetera, the banks, they all had copies. I made sure of that. So doing as much documentation as soon as you know your loved one has dementia—- is absolutely critical. The more admin prep you can do ahead of time, the—- easier end of life will be. I c—I cannot stress that enough… That I didn’t have to do any of that in my crisis situation was a huge benefit. (23)  but even though I’m the youngest and the philosophy, da-da-da, um, the folks knew, you know, I was executor for their estates and-and all of that. And so, uh, and it—and my older brother and older sister uh, one, my sister’s an attorney, you know, they understood it. And that-that worked well to uh, to uh, uh, when—I was with my folks when they made their wills, but at that time, my father’s health was pretty darn good. And uh mother, I don’t think there was any indication of uh, of the Lewy body setting in. That was well in advance of that. (24)  He developed pneumonia, and at that point, um, they started to give him antibiotics. And I’m thinking of my—of the—the advance directive that he and I both filled out as soon as he was diagnosed, um, when he could still write his name. We, you know—we did the will, the—the living will, you know, and the—the power of attorney and all that kind of stuff. So, um, you know, at that point, as we said in that, you know, not—no heroics, and under the heroics, um, antibiotics was one of them. (25)  We had our—all of our deaths and burial and so forth, stuff all taken care of a few years back, so I didn’t have to deal with that. (27)  **Patient being clear on wishes**  He was always very clear about what he didn't want. You know, he didn't wanna ever be put on a ventilator. Um, he didn't want a feeding tube. Um, so that helped, you know, to make our decisions a lot easier because we knew exactly what he wanted and what he did not want. (1)  He was pretty vocal about, um, not extending his life past, um, um… He was very clear (5)  we had already discussed what happens with the feeding tube, and he had—I-I knew he would, you know, he-he would not want that. (5)  he made sure all of his children—so between he and my step-mother, the eight of us, every one of us was made fully aware, here’s the situation. {Interviewee’s Name} in charge of the finances if we both go. You know, it’s gonna be split equally. You know, if I need to be put on tubes, I’m not gonna be put on any pipes or tubes. I just—I want—I just wanna pass away. Um, so he was pre—they were very clear on that, on what they wanted done. (10)  he gave us a clear, uhm, [distorted audio 15:10 – 15:14], you know, what each other wanted in a situation, you know, surfaced if that came if they don’t want like any type of, uh, life support as far as like you know, a ventilator or anything like that, but he, uhm, he-he told us—he told me—and I just felt so bad—he just told me when he was at the geriopsych ward he did not want to go to a home. I don’t—and because he would come in and out. (19)  I kept my husband home. That was one of the things he said early on, “I don’t wanna go, I don’t wanna go to a nursing home,” and I committed to him. Um. He won’t go. And that’s, that was really important to be home I think. (21)  you know, we knew what he wanted, which was nothing, you know, no—- uh, um, no measures to extend his life. (22)  It was hard, and with the advance directives, he—my dad always says, “I don’t wanna be left alo—on a tube or anything.” Growin’ up, he’d always say that. And, “I don’t wanna be laid out in a coffin so people could see how good or bad I look.” So, we knew about that, you know and… thought that was pretty gruesome and stupid at funerals, you know, when that would happen. But, um, you know, he said, “I don’t want that. I don’t—I just—closed casket,” you know? So, we knew about that, and my brother, he couldn’t really talk to my dad about want he wanted because my dad denied that he had dementia…. But, yeah, we-we did know he didn’t wanna be on the tube, and I felt comfortable sayin’ no tube feeding or anything like that. I felt no-no guilt or anything cuz I knew that’s what he’d want. He was ready to go. (29)  **Family discussing whether patient wishes were right**  He was very-ver—he-he would have been very, um, um, specific about DNI—DNR orders, and there was some discussion among the family about whether that was right or not. (5)  **Benefit of having discussed before the patient got sick**  Well, it was a time of saying goodbye. Uh, at that point, we had made all our decisions. We knew what was gonna happen. You know, we had talked about it before he got so sick, and, you know, it was—we didn't have to worry about all that, because all those decisions, per se, had been taken care of. A-again, I—well, I'm an advocate for patients taking care of themselves, you know? (4)  **Difficult asking patient about end of life wishes later in course**  this whole thing with end of life and the feeding tube. Even though it’s in the will, even though it’s, it’s written out as a—what is it called? A, a DNR or something? It’s, you know, you have to say to the person you know, your loved one, “Is that really what you want? You know—“think about it.” …And so you can give an example. What if you aspirate? What if you have to have a feeding tube? And what if you get C Dif because you’re on all these antibiotics and everything. Do you really want to continue living, you know, no matter what the quality of life is? And you might, you know it’s kind of cruel and that’s where you need a second person in there to kind of facilitate. So that what happens if you can’t walk, you can’t talk, you can’t go to the bathroom, you can’t, you know, see? Um, because I really don’t think he was seeing. (21)  **Caregiver advocating regarding pre-expressed wishes**  We want to bring her home," um, because that's what she would want. (3)  {Husband’s name} didn't want—he—any further test. They did an EEG. They wanted to do a MRI, and I absolutely didn't want them to do that. (4)  he was gonna be cremated and not embalmed. He did not want those, to be embalmed. (21)  The thing that I did for him that I know he wanted was not to cause him to, you know, suffer by virtue of, um, medical interventions that weren’t gonna do any good. (22)  **Changing mind about decision**  He, he couldn’t tell me what he wanted. There was no expression on his face at that point either. Um. Because when we put the feeding tube in, uh, two years previous I asked him, you know, he, it was sort of a, a shock. He had asperated and, um, uh, you know, uh, and in his, in his will or, yeah will I guess, is that no extreme measures should be used, but when I asked him, you know, I told him what was going on. I talked with our doctor. And you could tell on the look on his face that he did, he wanted the feeding tube. So there’s that issue now there was no expression. You know he had deteriorated to the point, um, you know, what do you do and so emotionally as a, as his wife and caregiver. - I had to make that life and death decision for him (21) |
| Family Discussions about end of life planning | **With family**  what they think is that they're going crazy. I mean, that's what my dad said. Um, you know, he'd say, "Would you tell me if I was going crazy?" And I said— Dad, you have an illness." And it- it- it- it's very, very sad. (9)  …he made sure all of his children—so between he and my step-mother, the eight of us, every one of us was made fully aware, here’s the situation. {Interviewee’s Name} in charge of the finances if we both go. You know, it’s gonna be split equally. You know, if I need to be put on tubes, I’m not gonna be put on any pipes or tubes. I just—I want—I just wanna pass away. Um, so he was pre—they were very clear on that, on what they wanted done. (10)  When we had our big family conference, you know, it could have helped them understand a little bit more. (17)  **Different levels of understanding within family**  And over that time, and I kept telling my mother, we had weekly, um, conference calls with my mother, my sister, and I. I would go down to the unit and sit with the doctor and the social worker and then we would all be on a call together. And he would describe what was going on and- and my mother said, "Well, {Name} is- is explaining this, but I think she's just being dramatic." And— the doctor said, "No, she's being really accurate of what's taking place now." (9)  I knew when she no longer could eat there was no way I was gonna do a feeding tube or, um, but it’s all those types of things that I was prepared being a physical therapist myself, but my brothers weren’t. Just people that I talked to in general they’re just not prepared about what the end is going to look like. (13)  We had a family conference before he came home from the hospital, after he had had a stroke. And it was because his family—his sons were so angry at me, because this was all happening, 'cause they did not understand it—- even though I tried to tell 'em. Um, and a lot of the talk was about the stroke, more than it was about the Lewy bodies. (17)  **Challenge with different opinions within family**  we had talked a—my father and I had talked about um—he-he had mentioned for many years before he died, “I wanna be cremated.” And I said, “Dad, do you think you might wanna donate your brain to science?” And he said, “Yeah, that might be a good idea. Sure, okay.” I said, “Okay.” But those were conversations that we had in my car, like I am right now, um without another witness. Uh, when the time came, I said to my mother, “Hey, look, you know, we can help a lotta people. Why don’t we donate his brain to science?” “Absolutely not.” “Okay. Uh, you know, Mom, he wanted to be crea-cremated.” “No, no, no, no, no, no. We’re gonna have a casket.” (23)  **Regret that ACP not done**  I mean him and I did a lot of talking while he still had his memory intact yet - and de—de—decide what to do. And I wish—I really wish that we would’ve talked more about it before he was too hard to talk to. (11) |
| Family Discussions at end of life | **With providers**  And over that time, and I kept telling my mother, we had weekly, um, conference calls with my mother, my sister, and I. I would go down to the unit and sit with the doctor and the social worker and then we would all be on a call together. And he would describe what was going on and- and my mother said, "Well, {Name} is- is explaining this, but I think she's just being dramatic." And— the doctor said, "No, she's being really accurate of what's taking place now." (9)  And that day we had a conference call with my mother and the doctor told her that she had to come to {Indiana}, that there were hospice decisions to be made (9)  You know, we talked with {Doctor’s name} and had a meeting and, um, we're Catholic and so we, um, had the priest come and give him the last rights. (9)  the conference calls were really helpful. we had had weekly conference call- conference meetings when Dad was in the rehab facility in {City Name}. (9)  we—in the course of a conversation about that, you know, regular care conference, the social worker asked if we had considered hospice care for her. (19)  So, when he, {Husband}, was released from the hospital, um, I called Dr. {Name}, and he came to (Name} Village, and we met in the library. Um, my s-son, my stepson, had come from {Washington State}, and, uh, my stepson, was on, um, a conference call. And we met with him. And it was that trust, and he explained to me why it would not be advisable to keep him on, um, th-the antibiotics, which— actually, the doctor gave me the antibiotic, um, um, prescription just because I-I was in shock. I said, “No, no. You can’t stop it now.” [Laughter] You know? And he gave us the alt—you know, he—it described the alternatives for hospice and, um, not. Um, and so, w-we chose hospice thinking, at that point, that he—I was hoping that he would graduate out of it. (26)  **Sub theme: Regrets about not having planning:**  along the continuum of her care, yes, so I would’ve had, um, a—a like kind of mediated process for identifying important transition points well in advance of when they happened. So what can we all agree on would be, you know, the terms around which she’ll move to assisted—or the—into the lock—the lockdown unit, that I called it…what can we all agree—you know, just having those conversations, uh, well in advance would be really helpful (2)  had-had we been better prepared, I think that we would have been able to do things in a way that we all would have been happier with. (3)  in hindsight, I really wish that, at the very beginning, at the get-go of when things started to unravel and go down the steep decline, when we started hospice, I wish that we had done a conference call….had-had the hospice doctor explain to all of my siblings, because they were out of state, or even if they were in-state somewhere, at a jobs—- or whatever, and explain what was going on with her, uh, where she was at, what to expect, timeframe, that kinda thing, if they wanted to-to come out and see her. Um, instead, you know, the—I get the information and it’s—as a caregiver, you’re already extremely like fatigued [laughter] and—- and it’s hard-hard to remember all that. And, um, it’s just overwhelming. And so, it would have been better, uh, I think, had we done that. I had a sibling that was in denial that my mom was gonna die. - I—you know, it-it would have—that would have just helped to, one more thing, to say yes, this is true. (8)  I could have talked to them, or the doctors could have talked to them a little bit more about Lewy bodies, had they been more understanding of what all the symptoms are - or can be, and that this is all probably related to the Lewy bodies, you know? When we had our big family conference, you know, it could have helped them understand a little bit more. (17)  …the negative of mother—of honoring mother not wanting to know is that her children never got to discuss it—her passing with her. And uh, and, you know, crud. But, it really is all about honoring her wishes. (24)  I don’t know, because I’m still in the mid-middle of it, what would have happened—what could have been done any differently with the, um, rest of the family. And they took it so-so personally that I didn’t call a big family conference— to ask where he should go in all this. And it was a matter of I couldn’t handle it. I had—there was an opening. I had to do it now. And if I had asked each one of ‘em, we might still be here trying to - to figure out because each one of ‘em would have—yeah, each one of ‘em would have had a different idea. (30) |
| Decision making about details for after death | He was. He was. I mean he definitely had—had a—obviously had a cognitive, um, deficit, but— we could carry on a conversation, and he was still, um, able to give us his opinion when—you know, he agreed to have the procedure. Um, so he did participate. Yes. (1)  *When asked: was patient was still participating in the discussions about his medical care*  … I had his funeral paid for three years before he passed away. And he didn’t—he didn’t know it. I just asked him when he was still a li—little bit—he knew what was going on. You know, I said…, I just said to him, I said, “{Brother’s name},” I says, “What do you want me to do with you when you pass?” You know, and he goes, “Well, I wanna be cremated, and I wanna be buried above Mom and Dad.” And I said, “Okay,” and it—that was just so simple… something so simple like that that a fam—family members can do, so it’s not a burden at the end… everything just went so smoothly after he passed, it was unbelievable cuz I had everything in order. (11)  we had talked a—my father and I had talked about um—he-he had mentioned for many years before he died, “I wanna be cremated.” And I said, “Dad, do you think you might wanna donate your brain to science?” And he said, “Yeah, that might be a good idea. Sure, okay.” I said, “Okay.” But those were conversations that we had in my car, like I am right now, um without another witness. Uh, when the time came, I said to my mother, “Hey, look, you know, we can help a lotta people. Why don’t we donate his brain to science?” “Absolutely not.” “Okay. Uh, you know, Mom, he wanted to be crea-cremated.” “No, no, no, no, no, no. We’re gonna have a casket.” (23) |
| Families not wanting to let loved one die | she just wanted him to stay alive…. He had to have, um, Heimlich maneuver done on him many times as his smooth—smooth muscles deteriorated (2)  at one point the one—the—actually, the—one of them, the—the scientist one said, "Well, don't we need—" I bring the hydration up. "Don't we need to give him, like, hydration? Shouldn't we put him on an IV." And so then that's when I went into "No, this is—you know, this will cause him more pain if you do that." (18)  Why is he here? He’s DNR. No, we can’t admit him; he’s DNR.” But what good does that do? I’m going, “This is a human being, and he is obviously ill.” And, um, we went round and round. And then, of course, they just left us sitting in the ER for hours before a doctor finally said they would admit him to give him the IV antibiotic…. It went well the first time he was there for the dehydration - and having real problem, except one of the nurses, um, was determined to send him back home as soon as possible. And I’m like, um, you know, I can’t-I can’t move him, [laughter] you know? Um, and she didn’t care about that. The doctor was willing to admit him overnight to make sure he was fully hydrated and was, you know, back to what his current norm had been. But, nope, the nurse overrode that. (28)  ***Trying to feed until very end***  It was awful. A-and, um, and-and I had to say that because people cheated. You know? People fed him. (5)  he had to a girlfriend at the time. She would come for breakfast and she would handfeed him. Um, and that was for her ow—you know, that’s—that was—that’s consoling yourself… and then nurses that would forget or people that would eat in front of him, or bring him a treat. (5)  but he stopped really wanting to eat and it lead to, you know, him being fed. And I ended up feeding him all three of his meals every day, um, and encouraged him and little by little still he wouldn't eat them. And at the very end, he was, you know, choking, you know…(9)  The last night that he spoke I was trying to get some Ensure into him. We were trying pureed foods. We were trying everything. (9)  but he stopped really wanting to eat and it lead to, you know, him being fed. And I ended up feeding him all three of his meals every day, um, and encouraged him and little by little still he wouldn't eat them. And at the very end, he was, you know, choking… (9)  I wasn’t overly concerned if I couldn’t be there every day to help, you know, feed him, because, you know, towards the end he was at a feeder table and everything else.  He would get so mad sometimes because he—he wouldn’t eat, and then I would try to feed him. And he would kick at me, or he’d yell at me, you know—- and tell me we never come and visit him, and I was there every day, you know. (11)  But she-she-she started takin’ less and less on, um, and then we got to the point, where she was maybe just having a couple of sips of juice a day—- a little bit of pureed food and perhaps a yogurt. And then the day—and-and-and it was gettin’ more and more difficult to get her to-to take stuff on. And then I was-I was—a-and-and—but, again, they were almost expecting me to do it. And by that point, she started to make this, uh, kind of gurgling sound. And I was really uncomfortable feeding her by that point because I-I didn’t know—I-I didn’t really know what-what was choking and what wasn’t. I-I didn’t feel as though I was able to determine whether it was going down properly or not… But, um, you could-you could hear a definite gurgling sound, and she said, “Right, I think that—I don’t think she’s-she—I think that’s gone on to her lung. I think we should stop.” Um, but I do feel then as though the—it was almost as if the—it said, “Right, that’s it. She can’t swallow anymore, and we’re not even gonna try.” And I actually went to a nurse and said, mmm, “Well, what happens now?” And she said, “Well, nothing ‘cuz, you know, she can’t swallow. She can’t swallow.” And that-that just seems to be almost the end of it. (16)  **Are decisions for patient or family?**  explaining the feeding situation was grueling. And what drug it out—well, I-I really don’t know what drug it out so long, other than we made the decision, and he had to a girlfriend at the time. She would come for breakfast and she would handfeed him. Um, and that was for her ow—you know, that’s—that was—that’s consoling yourself. (5)  she was like, "Well, can't we put him on fluids? It just seems cruel." I said, "Does he look like he's suffering?" "Well, no." It's only to make yourself feel better and he had signed— years before what his request was. I said, "Please don't do this to him." I said, "He is not going to survive." (9)  there are all these issues like tube feeding and ‑ and medications, and should you give this or that to prolong life? Um, and I talk to my people in the Facebook group about, you know, are you doing this for him, or are you doing it for you? And there is a process to letting go that you have to allow yourself. (15) |
| Providers not wanting to let patient die | there were a lot of great care providers who, um, loved her and, you know, were tender with her. And that was—that was, of course, good. Um, there was a hard, um— the woman who loved her most that we adopted as a fourth sister, um, she couldn’t give up on, um, wanting her to live. And so then there was tension around that— And, um, she ultimately, um, just stopped coming into work for a bit. Her back went out, and I’m sure it was stress-related because we were there trying to facilitate my mom being able to die, and she was trying to fight it. (2)  Doctors are trained to extend life - whatever they need to do. (6)  And when my wife was in the memory-care facility—and we’ve seen this with other members there, patients there—the minute they stop eating, they stop drinking, they get to a point if they don’t have hospice, if they don’t have specific requirements, right away they take ‘em to the hospital. They feed ‘em intravenously because that’s what they’re trained to do,— and then they bring ‘em back, and then they go through that cycle again; (6) |
| Patients not wanting to live with dementia | “And as soon as they said dementia, I-I believe my father called a hospice service to meet, um, at his home. Um, before—a-a-and then, for us to be talking about—care. Um, and as a—as a lifetime polio survivor, he had some really strong feelings about euthanasia issues, and-and that was probably one of his biggest fears. So, he made a call to a—to a local hospice program, um, who just came out and had a talk with us (5)  it’s kinda questionable what-what-what quality of life is, but he-he-he lived it, you know? And he—…he decided when he wanted it to be over, and it was not over for four, five more years. (5)  Dad had, in my mind, made a decision that this wasn't gonna get any better and he wasn't gonna hang around for it. And so his condition deteriorated the entire time he was there. (9) *Speaking of dad’s attitude about dementia while in facility*  her life partner of 25 years had passed away about 3 months before her. I think that that was part of why she just wanted to leave. But, basically, she stopped eating. She refused to take her medication. Um, and she was going into much more hallucinations and just very, you know, out there, yelling and upset. (3) |
| Patients desiring death (passively) | she just started—she had been saying, for a while, that she just wanted to-to go to heaven…- she had been saying for-for quite a while. (3)  I think he—you know, he had already decided—as-as a—as a paralyzed person, he had already decided that, if your quality of life s-stunk, you know, it’s over— (5)  She knew it was coming, the end, and then she started shutting down. And she um decided not to want to eat anymore. (6) |
| Recognizing end of life coming | He knew that he was getting worse and worse, and we knew that, you know—we knew he was gonna pass. It-it-it wasn't a matter of, y-you know, wondering about that. We-we knew that. (4)  If anything, it might be a grunt or two, but nothing beyond that. She knew it was coming, the end, and then she started shutting down. And she um decided not to want to eat anymore. It started out by eating just a little bit,— - down to absolutely nothing. (6)  My wife would never wanna know she was gonna die. I know that. She felt—I-I-I’m certain she felt she was gonna die, you know, soon, but I don’t think she wanted to hear that. (6)  she never really woke up after Monday. She-she slept, didn’t really respond to me. Wasn’t—I could tell she was there, but, you know, she hadn’t crossed the line of no return yet. But there was a difference…. And I just—I said, “Look, {Caregiver Name}. I think we—I think we’ve got her for about two hours.” And two hours later, she was gone. (7)  it wasn’t maybe a—you know, as-as painful as it could’ve been. I mean you-you kinda did go through sort of this, you know, wow, quick, it was definitely a progression that you could see, you know, the end was coming. It was going to be over the next few weeks. (10)  I guess we could also see it coming because he lost his-his abilities to ambulate had actually dropped off. Kind of, you know, in conjunction with the appearance of some of these other end-of-life things that were happening. (10)  it was very hard for him to swallow, so we made the decision of getting him off of everything and..He seemed to be okay, but you know, we knew it was—it was getting to the end. (11)  So she was saying things like, you know, his cheek—the skin on the cheekbones was tightening, and he was—breathing more, uh, ragged, and he was, you know, sleeping all the time. You know, all of those kinds of signs that‑ the end was coming. He didn't want to eat at all, even pureed food (15) *Speaking of nurse who ran home recognizing end*  I had a care, another caregiver that was so wonderful. He had a lot of experience with nursing home patients. And he was a private care also. And he, he knew a lot about end of life, looking at the person. He said to me when he left that night, “I think this is the night {Wife’s name}.” Because he showed me his, his, his pooling and, you know, the blood, and the skin, and, um, but my husband was in such good shape with his heart that he was not heavy breathing until the very end. I mean the very end was I woke up and I was about ready to give him morphine again and he—- was just breathing *[panting 18:59]* and I, and I ran. I got panicky and I got the morphine and he, and he was gone. That was it. (21)  what’s interesting is uh when hospice came, they started looking at his legs and the um um—oh, what’s that called when the veins start showing? Um, I forget what it’s called. Anyway, when you know that you’re passing (23)  And I had talked to the-the evening shift, uh, doc—uh, n-nurse. And he explained what I would see. You know, the legs— and how they become, uh, reddish and blotched and, you know, he explained some of the things. So, we went through the night. And the next morning, he was—uh, it was obvious that he was very close. And the nurse said to us at about 7:00… He died at 9:16 that morning. Um, she did not leave. She ended up staying, I think because she knew he was dying. (26)  i-it was so shocking, because he was foaming. Th-there was stuff just coming out of his mouth. And I didn’t know that’s what would happen. I just had no idea. And they were suctioning him. And what happened, apparently, is that his lungs had filled up with such goop, or whatever-that w-when the nurse came and turned him for the bathing, and they repositioned him, it starting coming up. And I knew then that that was—that was the end. It was— the end. (26)  **Giving the patient permission to die**  ”… And he said, “I think this is the night {Wife’s name}. I just have a feeling.” But he came and he told my husband, and it was just amazing, he said, “{Husband’s name} you’ve worked,”—he was a physicist [voice cracking] and he worked in fiber optics. And he said “{Husband’s name} you have worked in light all your life. Go to the light.” And my husband died about four hours later. So it was really amazing. (21) |
| Prolonged “death watch” | It was just really painful, and she was ready to go, it seemed, although she still wouldn’t die. I mean I really learned a lot about how hard it is to die. It’s just—so she went—it was three weeks where we basically sat deathwatch on her. And for five—the last five days, she didn’t eat or drink anything. Yes, so excruciating.(2)  I mean she just should’ve died. It was just, you know, she should’ve died many times over, and so that was sort of bizarre (2)  Um, but I didn’t realize how long that would take. And it was—it was awful… - they told me it would be about 10 to 14 days, but it ended up being more like 3 months. (5)  And what drug it out—well, I-I really don’t know what drug it out so long (5)  a couple nights I stayed over with her, and one-one night they—you know, [inaudible 11:15] call every couple hours and—to like, “Maybe you should come back.” So I-I went back there, and they said she’s kind of—really labored breathing. And I s—I-I sta-lay-laid there and counted. She was breathing every 40, 45 seconds. It was crazy. (It was really, really hard, and I literally was up all night, you know, count—uh watching the clock, counting the seconds tick off. And I’m like, is this it? Is this it? Is this it? And then she’d go [gasps], and then we had another, you know, 30 to 45 seconds till she did it— again. It’s crazy. … And-and then you-you hear these things like, is it that [clears throat] she’s waiting for me to go away so she doesn’t see it, or, you know, what does Mom want? Right?.. Maybe this would be the opportunity that I’m not here; she would go. So I stayed away for a couple days, and that was—it wa—I-I don’t—I don’t know. I’m not sure if it was the best thing to do or the wrong thi—I don’t know, but I did, because I needed to rest, and I needed to breathe. Maybe this would be the opportunity that I’m not here; she would go. So I stayed away for a couple days,.. I took a couple days off, and she was still ar—still with us, (14)  while, you know, then because he was a very—he had been an athlete, was a very healthy individual, he was totally unresponsive, so for that, I—I don’t even know how many days it was, but it—it had to be a good week. No water, no, um, food, and he just hung in there. It just took a really—what seemed like a really long time for him to— No water, no, um, food, and he just hung in there. It just took a really—what seemed like a really long time for him to to come to it, so, anyhow, [laughter] next. That’s—but that’s the overall story. (25)  **Difficult to predict moment of death**  the doctors, of course, they don't know exactly when somebody's gonna pass away. So they're— - just kind of guessing and saying what they think. But, you know, so-so that-that was kind of how we got to where we were. Um (3)  but at night the nurses were very insistent that we go home and get a good night's rest. Could we stay there and sleep in a chair? Fine. But who knew how many days it was gonna be. (9)  She, um, stopped eating and I guess over, um, a period of about six or seven days she held on. Um, didn’t eat, didn’t drink and slowly, you know, went down. Um. And then did finally, it took a good two or three days where Hospice kept saying, “It’s gonna be any minute, any,” you know. She held on. And, um, that was the first experience that I had with someone actually that I loved dying. (13)  And um, a couple nights I stayed over with her, and one-one night they—you know, [inaudible 11:15] call every couple hours and—to like, “Maybe you should come back.” So I-I went back there, and they said she’s kind of—really labored breathing. And I s—I-I sta-lay-laid there and counted. She was breathing every 40, 45 seconds. It was crazy…It was really, really hard, and I literally was up all night, you know, count—uh watching the clock, counting the seconds tick off. And I’m like, is this it? Is this it? Is this it? And then she’d go [gasps], and then we had another, you know, 30 to 45 seconds till she did it - again. It’s crazy. (14)  her breathin’ was very shallow of—uh, you know, from-fro-from that sort of, like, five days onwards, her breathin’ got shallower and shallower. And we kept sayin’—because, um, my sister and myself were there, but-but her husband wasn’t. And we kept sayin’, “Is there any—you know, can you tell us now how long we’ve got?” because we’ve got all the family members that we want to-might want to get round. And what they kept doin’ is checking to see—uh, checkin’ her fingernails to see how blue they were goin’ as an indication of, you know how the—you know, there was—she was sufferin’ from, you know, oxygen—uh, um, lack of oxygen. And that’s how they seemed to be determine-determinin’ how long she’d got left, um, which I-which I thought was quite, um, you know, unsuccessful, shall we say. Um, but then, uh, the final day, um, uh, her-her-her breathin’ got shallower and shallower, to the point where you actually had to ke-keep checkin’ she was still actually breathin’. (16)  So, we would-we was-we kept going out to ring him to tell him what were going on but didn’t feel as th—didn’t—couldn’t say to him, “Right, you need to come now,” ‘cuz we never got-we never got to the point where we were told, “We now think”— - “you need to gather the family round.” We were never told that, even though we kept asking. Um, so we just kept, uh, uh, ringing-ringing him every half-day and updating him, and obviously because we were never told, “Right, we think now it’s hours. You need to get whoever you need here,” he wa-he wasn’t there when she passed. (16)  Hospice won’t tell you when they’re getting—you know, you, you always kind of, as a person you wanna know well is it now, is it—when do you think, you know? Because I know this sounds anal, but I didn’t want him to die [voice cracks 07:06] without him being dressed up and they made me fearful of not being able to move him myself because he was gonna be cremated and not embalmed. (21)  And so it’s, it’s very difficult knowing is this the night, is this the day, and you keep asking Hospice and they, they, you know, you know, there are certain signs, but no one knows. (21)  So I sat there, you know, 18 to 20 hours a day, and the other 6—4 to 6 hours I made sure my family was there ‘cuz I didn’t want him to die alone. (22) *Speaking of patient dying close to 3 weeks.*  so we-we worked together and um, but we sort of held vigil for the last couple, three days. Um, I remember that uh, the hospice lady had said uh, you might wanna call your brother and sister, and that was on Easter Sunday, which I did. But [Laughing] I actually, she said it. I-I didn’t—I-I—it was in—it was not the regular nurse… And uh, and she was at the foot of mother’s bed and she said, “Well, it won’t be long now,”… So, I felt like, because of my mother’s stubbornness and uh, independence, I felt like she probably, maybe somewhere, heard the hospice nurse say that. And so, it took her two more days. (24)  the main caretaker for her, a young Hispanic guy had just stepped out to run to the restroom across the-the hall. Just stepped out. So, in those few seconds, or minutes, cuz he came back in and she was gone. (24)  every night, I would think, “Maybe he’ll die tonight.” And the next night, “Maybe he’ll die tonight.” And then next night, maybe, so when it actually happened, you know, I didn’t really know when it was gonna happen. So the hospice people called me, and they said, “His breathing has changed.” Um, and it was about a half an hour away, but by the time we got there, he had already died. (25)  And my husband and I stayed with him. Not 24/7, you know, we’d leave and then come back and—but I knew it’d be three days, and I let everybody know it’d be about three days till hospice quit his pain meds and sedatives and started feedin’ him and this and that. Then it was like another three days and, you know, it’s kinda bad havin’ to see all that. (29)  And so I called and everything and the doctor said, “Stop any food or drink.” Uh, he said he’s not processing it, and he’s just gonna get in worse trouble with it. So that’s when we put him to bed and, uh, uh, well basically I sat there and held his hand for-for eight days. (30) |
| Actual death was sudden | But how he passed so quickly was just amazing. (4)  And then I could see he was dying all of a sudden, you know, and— I called the nurse. And I was able to, you know, hold him when he died, when he passed. And I-I could s-see stars in his eyes. (4)  we didn't experience the end of life as-as most of those patients did. You know, it wasn't, eh, dragged on. It was, you know— Well, it was—you know, the fact that he did pass when he did, the way he did, was really a blessing. (4)  I don’t think she or I knew um it was her end of life coming as fast as it did. That’s something I don’t think she knew. Uh, I didn’t, for sure (6)  my mom wasn’t actively dying for very long. (8)  truly didn't expect him to be gone that soon— and in that—and in that manner. (17)  the nurse practitioner came in and she said she didn’t think it would be that day. My dad was kind of progressing, but not, you know, uhm, and they—you know, we were kinda looking for what to expect, like the-the guy—like the last couple breaths they said it kinda like fish out of water breathing— but my dad didn’t do any of that. He took his last breath in and it happened to be, uhm, our, uh, our priest from our church came there to say a prayer with the family and he walked in and that’s when my, you know, my dad took his last breath. (19)  She just passed so gently and um, uh, again, I do—I-I-I’m not convinced that folks aren’t—have an awareness even at that point in their lives because the main caretaker for her, a young Hispanic guy had just stepped out to run to the restroom across the-the hall. Just stepped out. So, in those few seconds, or minutes, cuz he came back in and she was gone. (24)  And I looked at the clock, and it was 1:40, and I thought, “[Sighs] I wanted to be home by now,” and, [laughter] you know, so I just kinda sat down and-and was waiting and talking to a friend who was there with us. And she noticed that his stomach was no longer going up and down. And he’s on oxygen, so it’s not like I could tell from hearing his breathing that there had been a shift. But she noticed that she wasn’t seeing the movement. And I ran out into the hallway, and the doctor happened to be just down the hall, and I yelled for her, and-and doctor came back in. And she listened to his heart and said he does have a heartbeat. And within, you know, ten more seconds, that was it. So, it was like the announcement of, “We’re gonna get you home. The ambulance should be here. It’s on its way,” it was almost like he knew he didn’t wanna go. He-he was done because ten minutes later, he was gone. (28) |
| Easy end of life | And during those five days, they kept him, you know, very sedated. And, um, was- he was very comfortable. He only opened his eyes one time during that time and it was really literally hours before he died. They were peaceful (final 5 days) for us. I think that my mother, um, particularly would like to have seen- she didn't want him to be in pain or terribly agitated, but she would have liked to have seen his face animated once again. (9)  So the end came, uh, very easily, peacefully, quietly, just, uh, passed away in her sleep. So, that was, uh, a great blessing. She had been at the facility for three and a half years (12)  Anticipating when-when her last days was comin’ to have a pretty difficult time. But as it turned out, uh, blessed, in a sense. Uh, it just—- came quiet-quietly in her sleep one night, and that was-uh, that was it. So, your questions of, uh, could it’ve been, uh, better, uh, I could see how it-how it’s—well, it couldn’t have been any better or easier. It just went beautifully from our point of view (12)  even to, well, uh, two days before, we-we had—- her over to my apartment in a wheelchair. So, uh, the, uh, last days were still sort of easy for us. Uh, we were in close touch with the-the priest in our church, and he had stopped in, uh, a few days before. Uh, she had Communion and a few things like that, so it was, uh, pretty easy from that point of view also. (12)  I always felt that I did everything I could possibly do— - with the knowledge that I knew, but it was a—it was a good, peaceful ending for him with everybody around. I really feel like it. (18)  It was very peaceful. It was nothing, uhm, it was nothing stressful. I mean it was stressful, but, eh, given the situation— uhm, they made it as-as comforting as possible. (19)  So that all went very well. Um, I think the-the very end-of-life experience was gentle and-and as good as it can be. And I think for-uh, for {Mother in Law} they were able to do a reasonable job of managing her-her worst symptoms. We had established care priorities of minimizing physical and emotional distress. And that meant sometimes that she was medicated to the point where she was sleeping more than maybe was ideal. But if she was awake and crying all the time, that wasn’t [laughs] a good alternative. (20)  So I think in some ways that was comforting too that— - it wasn’t a, a hard death. It was a very, I think a very soft death. I mean - it wasn’t a, a hard death. It was a very, I think a very soft death. I mean - I didn’t hear a rattling or any of that. It was just a breathing issue. Um, and then he just, he just, that, that was it. (21) |
| Actual death experience | she was basically, um, uncommunicative, totally paralyzed, could barely move unless she was in some kind of pain, and then her whole body would just shake, um, you know, gasping for air the whole time. It was the worst seven days in my life and probably in hers, as well. (3)  Um, but then, uh, the final day, um, uh, her-her-her breathin’ got shallower and shallower, to the point where you actually had to ke-keep checkin’ she was still actually breathin’. And then about an hour—and then-and then, uh, a couple of hours before, it was just gettin’ really erratic and irregular and shallower. And then she actually stopped breathin’, um, an hour before she actually died. So she stopped breathin’. We thought, oh, she’s passed. But then, uh, a minute passed, and then she took a massive gulp of air, and then for an hour she was sort of, like, draggin’ air into her lungs and so badly that her jaw was cracking. And then she’d start breathin’ again, and then she’d stop, and then she’d start. And we were like—and the-and the nurse just said, “That’s-that’s normal.” Um, and it-and it wasn’t like that-that-that’s what we would expect, and it’s gonna go on for an hour if you need to prepare yourself. Do you want somebody with you or whatever? It was just, “That’s normal.” They went away and then an hour later said—came back and said, “How’re we doin’?” [Laughter] Like, well, she’s died now, but thanks. The last hour was pretty horrific. Thanks for asking. (16)  - but said, “At this point she’s comfortable. I don’t wanna put a cannula in her nose and make her uncomfortable”—, “At this point she’s comfortable. I don’t wanna put a cannula in her nose and make her uncomfortable”— And about 7:30, when, uh, the air hunger started, the-uh, the on-duty nurse at the memory care facility said, “We’ve gotta get oxygen for her.” So, it took a couple of hours to get it and then a couple of hours for it to really take full effect. But then she had a very comfortable night and, um, passed away the next morning. (20)  Um, back to the day he passed; um I wanna say his breathing changed sometime in the early morning, maybe about 6:00 to 8:00 in the morning, and he was breathing like that all day. Um, what’s interesting is uh when hospice came, they started looking at his legs and the um um—oh, what’s that called when the veins start showing? Um, I forget what it’s called. Anyway, when you know that- um uh that started to sh—and-and hospice pointed that out to me. And I-I kind of watched it happen the rest of the afternoon and whatnot. Uh, in the catheter, it was a very interesting different output, also; very, very dark, almost tar-like. Um, the day before, he had a very large output, and then the day of his death, practically nothing, but it was very—almost thick and lots of sediment, and it was very tar-like. Anyway, so uh at about uh six uh—quarter of seven, uh my mother, fortunately, was not in the room at the time. Um, and I say “fortunate” cuz I think that she would’ve dwelt on this. Um, uh he uh—he gave a number of very large breaths [breaths loudly], kind of gasping breaths, and then nothing, and then he gave a very, very large gasping breath, and his face went white. And that was it. . I was in the room with him. I was holding his hand. I—to this day, I’m so glad it happened that way. Um, and I’m so glad my mother was not there. (23) |
| Concerns regarding hopsice | we had hospice with my dad. He had end-stage COPD. Um, I had mixed feelings about-about hospice. Um, in my own family, I process s, and— I have a lot of trouble with that because I know that there are lots of people that, unfortunately, do it that way. Um, and I see that. I-I believe, uh, until life from conception to natural death. (7)  Hospice back then- 30 years ago was known, at least in my mom’s circle, for helping along, you know? You know? And so, she was terrified of hospice. (8)  And so I really fought not to put him on Hospice because I felt like he should have as much therapy and PT and—- good experiences as possible and not just let him have end of life experience before he was ready. So. That’s the thing with Hospice is I think that with this disease, dementia, the person should be allowed to go on Hospice but have the therapy and the exercise to keep their brain going as much as they can.(18)  **Perceived mistake by hospice**  ..my dad like failed like really quick where he wasn’t gonna eat or drink anything. He looked horrible. Just super stiff. And, uh, his face was all mask like and he was just kinda out of it. And, um, I told him, I said, you know, “Don’t feed him. Just, you know, let him go. I don’t want him havin’ any food or fluids or tube feeding or anything like that,” you know? And, um, he was dy—actively dying, and they were medicating him to keep him calm. Well, those idiots gave him a PRN pain medicine or whatever they were giving him to keep him sedated and clam. They ordered it PRN. So—- it had been two days without food or water, and, usually, it’s about three days, and-and they fall asleep and die. Well, I go in there, and, all of a sudden, my dad’s sittin’ up in the chair, and somebody’s feeding him…. And they thought he was fine, so they took him off the stuff that was dopin’ up to help him pass peacefully. And, you know, so then he’s sitting up, and they’re feeding him, and he’s eating, and they’re givin’ him a ton of fluids. I’m like, “Oh, great. Now, it’s gonna be another three days.” …for him to die. He was comfortable and everything, but it-it was—the hospice was really bad. That was a dumb move on their part. (29) |
| Dying with respect, dignity | we were all extremely comfortable with the idea that, um, my mom wanted to have the highest quality of life possible, and ultimately die as swiftly and with as much dignity as possible. (2)  We all truly were a team and it was to maintain my mom’s dignity and respect at home. (13)  so though even though he got the care at the facility, but the hospice there—they came in twice a week with a caregiver who showered him and shaved him and, um, did his—you know, did his personal care. That, for me—for me, that's another thing that I, um—I feel very strongly about, that these people should be given the dignity and respect that their hair is combed every day - and that they brush their teeth and that they—you know, that they give them that respect. - and that they brush their teeth and that they—you know, that they give them that respect. So the hospice did that. So the hospice did that. (18)  … I know this sounds anal, but I didn’t want him to die [voice cracks 07:06] without him being dressed up and they made me fearful of not being able to move him myself because he was gonna be cremated and not embalmed. And so I wanted to know when I could get his suit on him because [voice cracks 07:26] for me and I think a lot of caregivers this disease is so humbling. It takes everything away from the person. You know, they don’t have any control over their body at all. And my husband, I wanted him to leave this world looking dressed up. And good, you know, as best he could cuz he was, he was very, very thin. I mean when you moved him you had to move him so carefully because his skin was thin as, just his, he looked like a holocaust victim to be honest with you. (21)  but my husband deserves- and it’s not, you know, it, it smells. I mean you know— you, you don’t want that. You know, I mean at the very end when you know your husband’s dying or your wife you want the very best for them. And you want, you feel that inside. You want the respect that they deserve. (21) |
| “Right to die,” desiring euthanasia | “in the—the end, in the final case where it actually happened, um, she was in so much pain, um, she asked my sister, you know, the oldest one. One day, she asked her, “Can’t you just take me out back and shoot me?””, the next night, she said, “Can’t you just go in the kitchen and get a knife and stab me?” So it wasn’t fun anymore. (2)  I just think we shouldn’t treat animals better than we treat humans, or we should treat humans as least as well as we treat our animals. (2)  we were all extremely comfortable with the idea that, um, my mom wanted to have the highest quality of life possible, and ultimately die as swiftly and with as much dignity as possible. (2)  I mean it’s hard to kill someone. I researched, you know, every way I could think of to help her die… what if I gave her a bunch of Adderall, or what if I gave her, you know, a bunch of, you know, Xanax, or what if I—just, um, it feels—you feel so desperate (2)  I guess, lobbying for changes so that Minnesota has more Oregonian—but that doesn’t even work, right? You—with someone with dementia, you just never can take ‘em out. (2)  let’s find a cure or figure out a way to end—end our lives with a little more active human—humane ways. (2)  he would say, uh, “Can we call Dr. Kevorkian?” he would say… He would say, “Can you call Dr. Kevorkian?” and that was pretty horrifying to the staff at the—at the nursing— at the nursing home. And-and even after he completely lost his memory, he-he’d have them call and say, “Call that doctor for me.” (5)  just because it’s, um, um, when you have the—when you have those feelings, those, uh, end-of-life, um, death-with-dignity feelings, you don’t pick up the phone and call a Catholic hospital. (5)  he was, and I’ve become, a-a pretty strong advocate of death with dig-dignity, and-and— (5)  We-we treat—we treat our pets better.” Um, and I know that’s a very touch-touchy euthanasia issue, but he felt strongly about it with good reason. (5)  because his quality of life was-was zero. Um, so, um, and getting that diagnosis is not the time for—to be debating over it. And it’s—um, I don’t thi—I don’t think I met a person on the care-care team that could actually understand his unique position on it. a really strong understanding of quality of life. (5)  It was horrible… And-and, um, he would request a pillow, and I knew that that meant. Um, but I would say things to him, like, he would say, “Call the doctor,” and I’d say—I’d say, “But is it today?” and he’d say, “No, it’s not today.” (5) |
| Patients not appreciating family help | He would get so mad sometimes because he—he wouldn’t eat, and then I would try to feed him. And he would kick at me, or he’d yell at me, you know—- and tell me we never come and visit him, and I was there every day, you know. (11) |
| Glad when the end arrives | but it was a blessing and it was beautiful and one of my good friends said, um, you know you’re privileged because you came into this world in your mom’s arms and she went out in your arms. (12)  And that’s when she took her last breath, so it was beautiful. But, um, so the brothers were all here at the end and they understood it. (13)  but I know she’s in a better place and, um, you know, but it’s just, it’s a horrible, horrible disease. (13)  the whole going through-through dementia and learning so much about it, um it was, yeah, the worst experience of my life, but I was- also very pleased to have been able to be there, and be with her, and holding her hand all the way through, and especially at the end of life when I was—I was with her when she passed away. (14)  that was it. Mom was gone. So she went doing the uh—she went the way I think she was good, with her surrounded by her best friends, so. So it was good. (14)  We were grateful that they didn’t go on, that her life didn’t go on any longer than it did- because it really wasn’t—she wasn’t having much enjoyment from life *any* longer. (20)  he gave a number of very large breaths *[breaths loudly]*, kind of gasping breaths, and then nothing, and then he gave a very, very large gasping breath, and his face went white. And that was it. I was in the room with him. I was holding his hand. I—to this day, I’m so glad it happened that way. (23)  And uh, so there I was um, just still, in my mind, telling her um, you know, thank you and you’re loved and dad’s waiting for you, but thank you. We’re grateful. We’ll carry on. Not saying it. And un, her little breaths were very, very shallow. Um, and she drew in a breath, and-and then did not exhale. And she just passed so. I mean I would love to die that way. She just passed so gently (24) |
| Fear that death wouldn’t come quickly after decision, readiness | when it got to that point, there was underlying fear that it wouldn’t be it yet, and it was a fear because she was really ready to die finally. (2)  It was just really painful, and she was ready to go, it seemed, although she still wouldn’t die. I mean I really learned a lot about how hard it is to die. (2)  I don’t think a lotta people realize the distance between a decision and a d—and an actual death. (5)  . …he decided when he wanted it to be over, and it was not over for four, five more years. (5)  ..didn’t even know it could happen—it was a nine-day death process, right? It was a nine—nine days from when she took her last sip of water till when she passed - away. So also, not—n-no knowledge of the fact that it could take that long— was also cri—pretty painful and uh an eye-opening experience for me. (14)  also mother, physically, was in pretty good shape. Uh, we had a massage therapist. I mean I think that her mind was dying and-and probably someone who was not as in shape as she was— who didn’t have, you know, two hour massages three times a week um, um, you know, but to uh, to-to being in the pool um, because I remember, at the very end, checking her extremities for um, oxygenation. And uh, and I remember—I remember kind of laughing and crying cuz I said, “Wow, mother, you uh, phys—you know, you’re working out. You always told us to work out, and you worked out, and it—it’s really—,” her body just was pretty strong. The circulation was still pretty good. (24)  *Speaking of looking at mother on hospice two days before her death* |
| Effective Therapy/Creating Special Moments/Quality of life | Wasn’t that lovely how our family friends came and read you poetry last night?” (2) *Caregiver speaking to mom*  so she’s just a nature person. She loved to be outside, so we could, you know, kind of drop her into that, and we could her outside and go, and, um— (2) *speaking of need for adult jogger*  …my wife had been a church organist and choir director for, well, 50 years, so she was very active in-in music. And, uh, the therapist would—uh, it was amazing to see how she would, uh, come to life with what the music therapist was doing. She’d be almost, uh, uh, out of it but would, uh, very much come to life. (12) *Speaking of music therapy*  So, occasionally we arranged to have the, uh, wheelchair van bring {Wife’s name} and her caregiver to my apartment— they—for-for lunch in the afternoon. So, on—uh, this turned out to be, uh, two days before she passed away. Uh, we arranged to have her come to my apartment with the caregiver, and we were watching the Olympics on television. She’s very much alert and attuned, and she’s, uh, unable to speak at this time, uh, other than a few words, uh, yup and nope. Uh, it’s hard for her to put two words together at that time. But, uh, she was clearly, uh, enjoying this, uh, um, television program. (12)  Through Hospice, we did some art therapy when we could We did, um, a woman who played the cello or violin or something came in and played for her, but, um, uh, you know, I, I couldn’t have done it without Hospice. (13)  the girls—all of us would be always holding the han—his hand or, you know, touching his head and, you know, taking—you know, that kind of thing. And we—you know, we were playing music and singing to him and just keepin' it all very peaceful. And he didn't once—at one—at—and—and my daughter that was there for him the whole day—she said the same thing, that there was no, um—the only time he would get upset was they, of course, come in and have to change his—- uh, you know, for incontinence. (18)  Uh, she had weekly nursing visits until it—except in those troughs, when she was seen more often and, of course, daily at the end. She had music therapy at least every two weeks, massage therapy every week, sometimes twice a week at the end because it was her favorite. She had two whirlpool baths a week, and, um, she had a hospice visit her who was, um, there every week throughout the-the year she was on hospice, read to her in the beginning until she could no longer really understand or-or hear. Uh, so that was a huge help to her and to us because it gave us a-a little relief. (20)  I do think he was peaceful. He, he… I don’t know how much he—I think he heard and I had music going all the time. Cuz he was, you know, he loved music. I had very soft music. (21)  the other things with the, the healing, the priest, the guitaring, the playing, they came, the harp she played it was, it was beautiful. (21)  they live in a one-level house, thank-thankfully, and he would spend most of his time either in bed listening to music—I bought him a little uh iPa—or iPod shuffle, and I—- loaded on a bunch of his favorite music, and he would go and he would listen to that, because I read somewhere that dementia patients love music, and he—- always loved music, so that was wonderful. Or else he would sit in front of the TV, and he would watch his favorite um stock market shows. He loved to watch stuff about the stock market, or Shark Tank; loved-loved the Shark Tank (23)  We had to lift him up and put him in a wheelchair to be transported. Um, a friend of my mother’s came to visit uh from out of town, and who—she’s a nurse, and she was very wonderful. She and I could lift him up together and transport him, and then lift him into the car and transport him to a-a restaurant. We all had lunch. He had a Diet Pepsi, and then we, you know, transported them back into the car and whatnot. Um, the last time he left his house for that was probably two weeks before he died. So what’s-what’s amazing…, my earlier comment about the h-ox, uh being like an ox, two weeks before he died, and he was still wanting to go out of the house. (23)  at the end um, she—again, hospice guided us with um, um, uh cuz we had we-we-we had been taking her bed out to be in the sunshine—- and uh, in front of the TV where shows made her laugh and uh, or, you know smile. Uh, but then uh, it became, you know, you’re—they gave us a fantastic handbook. Oh god, it was so good. And we saw what the book said and what they were saying was that her world—she started preparing to pass. You know, even with dementia and withdrawing. (24)  Um, we had kept mother in physical therapy. You know, we had—she had a nice pool and we’d get her in there with two others helping. And as long as she enjoyed it. We never—it was all—we were laser focused no matter what it took on her-her quality of life. Her ability to be—- to have any joy, no worry, no fear, only joy. And love. (24)  in the last few days, she started looking up-up and to the left in the corner of her room. And the hospice person said well, sometimes, you know, um, um, either it is—they are—either they are being visited by, you know, folks who’ve passed—loved ones who’ve passed before them, or they think they are. And so, you know, we slapped up all these um, we blew up pictures of our father and-and uh, and them together and um, uh, I mean we just did that throughout. When babies made her laugh, we found all these baby pictures and blew them up. And you know, you just—propriety kind of gets tossed to the wind as you do whatever. (24)  this gentleman um, uh, she caught sight of him outside cleaning the pool. And so, he knew her well enough, he acted like—and he did—he fell in. He hurt himself, even, falling in the pool but it made—it gave her a good laugh. Gave her a belly laugh.(24) |

| Hospice challenges | Well, what it came down to was that was the way we could get him morphine. And I had to literally drive like the wind to get to the pharmacy as fast as I could to get him the morphine, but we couldn't do that until ‑ until hospice was contacted and said, "Okay. Get him the morphine." So it was ‑ it was a madhouse. I was calling, calling, calling, and trying to get somebody to okay this fast. So then we did have a hospice worker come to the home, but, um, you know, what was she able to do for us? You know, it was just a preliminary look, and so we never really got any help. (15)  **Sub theme: No physician took responsibility for hospice**  I had called both of her doctors… And, um, talked to her family doctor, and, you know, none of them would make a recommendation for hospice. They said, "Oh, you have to contact hospice." Hospice says, "No. Your doctor has to write a order," un-until it got to the end. At one point, I said, "Get her into hospice. Let's just get her into the hospital, and we'll take it from there." But I had no idea it was gonna go the way that it went. So it wasn't—I mean, nobody can control it, but it wasn't the way I would have liked. (3)  And I don’t think enough physicians, um, just across the board and in general, you know, you think of Hospice for cancer patients and there is a definite need for Hospice and palliative care education for primary care physicians (13)  the doctor said, "We'd like to have a meeting with you." And she had a group of about four people, including a social worker, and they started out by saying, "Have you considered what would happen if your husband could not go home from the hospital?" Ka‑boom. No. [Laughter] Uh, and so they gave me 24 hours to find him a place to ‑ to stay. Nobody mentioned the word hospice. Nobody ever said it would be a good idea to look into that now too. Um, I didn't think of it. I should have, but, you know—and of course, we didn't know how end his near was. He was ‑ he was only two weeks in the home before he passed away (14)  We did inquire about hospice. Uh, none of our doctors recommended it. In fact, I called the neurologist, and he thought it was too early. Uh, [chuckles] this was in 2016 when he was really r-just really goin’ downhill, and I thought—- wow, it doesn’t seem too early to me. (27)  **Sub theme: Staff turnover/changes as challenge**  One of the things that I thought that Hospice should have done is they kept giving me different nurses all the time. And I thought that, that it would have been nice to have the same nurse, the same— continuity. Um, the same with aids. And I, I feel like Hospice who, you know, there were several different organizations I could have gone with, but I felt that Hospice should focus on their best aids for the end of life. Because this is the, the person with the end of life this is their end of the ride. They deserve that. Um, and sometimes I didn’t find that to be, like people didn’t really understand that you had to move people very gently. Um, because it was another, another, a different person maybe coming in every day so you had to start the whole routine all over again with them. And as a caregiver you didn’t have that energy. so I really think that Hospice should focus, you know, on making sure there’s continuity with the nurse and with their aids that they bring in. (21)  Um, so, I had 24-hour care. People came and went, and came and went. When they were very—the nurses, themselves, were very good…. And the nurse said to us at about 7:00, “Well, I have to leave at 8:00 and, um, and there’s nobody here, nobody to come until, um, the, um—until 3:00.”…. Um, she did not leave. She ended up staying, I think because she knew he was dying. (26)  And I found that, uh, in a situation like that there were too many people that he came in contact with, uh, and the—everybody’s way of doing things were different. And he had all his life been, um, a—oh, um, a-a fearful person…. He just didn’t have the experiences I did. So, um, he would give them a very hard time (30)  **Sub-theme: Uncertainty about who enacts plans/orders amongst different teams**  “once they figured out what to do, they couldn’t execute it across the team, you know, so it’s so many people. The hospice people take over for the doctor, and then the hospice people are communicating with the in-house people, but the in-house people are in so many different shifts, and— I'd call hospice. Hospice—says, "No. Your doctor has to write a order “(2)  **Family not knowledgeable about Hospice**  I think this is a—this is something kind of a key point, which you’re probably hearing a lot of; people don’t really know what hospice is. and they don’t know that it’s not necessarily—it doesn’t necessarily mean she’s gonna die in three weeks. (14)  But it—having an explanation from them about exactly what hospice would do differently than what they were already doing, I think that was the biggest problem for me, looking back—- is I didn’t understand the difference between the two. I thought they were gonna offer exactly the same thing - just with a different person because one is there because it’s a healing wound, and the other would be there because there wasn’t any expectation of healing. (28)  Speaking of difference between hospice and visiting nurse  And, um, what I learned much too late was the incredible assistance they could’ve been. Like, you know, I was payin’ for all of the, um, pads for the bed, and— um, the-the Pedialyte and things like that, whereas they could’ve been supplying that all that time. And I was already so stressed about money - and nobody said, “But they can give those to you as part of the program,” you know? [Laughs] That would’ve helped me so much on-on some of the fear about the financial side of it. (28)  **Sub theme: Hospice not familiar with DLB**  hospice I think would’ve been a little more in tune with the dementia side— - than a visiting nurse, not that they don’t have experience, but that’s not their focus. Hospice would have much more, um, insight into where he was and how he was changing. Um, if I would’ve understood—I mean, I was thinking that he was going to slowly lose his ability to walk, and then he would be bedridden for a while. And, you know, I’ve got this whole long-term progression in my head, and it wasn’t that at all. I mean, he was still walking, albeit not nearly as well, and he needed a lot more help, but he was still ambulatory, um, until the infection set in from the pneumonia. Um, that-that really made things more difficult to me to understand what was going on. And once he got the pneumonia, um, [clears throat] she had said, um, about getting food in him, getting something. And I said, well, you know, he—we knew that the thin liquids were a problem. And I said, you know, we’ve been doin’ these shakes that’re, you know, definitely thicker. She said, “Oh, yeah, that would probably be really helpful.” But then when I gave ‘em to him, I did not know that he was aspirating it. Nobody was there to say, “Ooh, stop. He’s aspirating.” (28)  **Sub theme: Positive hospice experiences**  “the minister was amazing… having chances to talk to her was helpful.”(2)*Speaking of hospice minister*  “I mean, it was just very, very nice, but it made it comfortable for the family. And me—and Mom's sister, my Aunt {Name}, we stayed with her 24/7. So they made it very easy for us, you know? They brought in an extra recliner, even though one—we were only supposed to have one person spend the night. Um, you know, there-there were—there was a place to go get food there. There was a nice, little courtyard, you could walk out into, just to kind of get away and clear your head. So the hospice was very, very nice. That-that-that was done very well.”(3)  “..and he was admitted to hospice. And, um, uh, he was very well taken care of there.” (4)  And hospice, you know, really took care of him and us. (4)  hospice services were great (5)  hospice, they understand that your loved one made a decision that she didn’t wanna live like that and live on life support stuff. “Let me go in peace.” So basically, they stop that process of a doctor saying, “Call an ambulance. Take ‘em to the hospital.” That doesn’t happen. (6) *Speaking of hospice*  hospice is a godsend. I cannot stress anything more than that… Hospice is there to make it comfortable because life is going; going to leave. (6)  and one of the things regarding hospice is, in my case, and I think in many, many cases, once they’re on hospice, you don’t get a bill. You know, that’s pretty well taken care of, I think—- by the state. I’m not sure, but that’s very helpful. They just come in and they really, really do help. Uh, oh, and also in hospice, they provide a social worker… and even to this date, the social worker calls me to find out how I’m doin’. Yes. Uh, they send me stuff, just bits and pieces of stuff, just to make sure I’m doing okay. And I can call ‘em anytime I want, and that is definitely helpful. (6)  I called them. It was 4:00 on Tuesday, and I knew immediately, the-the intake person was sincerely wonderful. [Laughter] Like, and very compassionate. I could just tell. And she—and she could tell I was—you know, I was upset, obviously. And-and she said, “Would you like me to give you a call next week, and do you wanna—do you wanna think about it tomorrow?” And I said, “No, I-I don’t need to think about it anymore. I-I-I’d like you to come.” And they had somebody there by 6:30. And they had a hospital bed in my house by 7:30. (7)  And I called and said that I wanted a hospital bed, and they asked me, you know, “Do you—do you still feel that you need hospice?” Cuz the doctor had talked to me— about it when a—when my aunt was in her last checkup before I left the offi—and she did ask me about hospice, as I mentioned earlier. And I was surprised that she brought that up at that point. And [sniffs]—but I left. I said I realize, you know, that, you know, she’s—knew what was coming and.. - you know, wanted to make sure I knew that she would—you know, whatever I needed. (7)  And so, she was terrified of hospice. So, I was trying to respect her wishes. Um, that was resolved easily by nobody said the word hospice at the end. She wou—they were just nurses and caregivers. So, um, in hindsight, I would have started hospice in July. I just was afraid to-to make my mom afraid. (8)  It was very peaceful. They were so good. They were so good. They, um—my brother liked country music, and so they had a lady come in there with a guitar and would play country music for him. Um, they came in every day and, you know, um, gave him like a sponge bath because he couldn’t walk anymore, and he couldn’t get up to go to the bathroom anymore. (11)  he just couldn’t do anything anymore. He just laid in bed, and they brought him a more comfortable bed, and they would, um, turn him all the time. I mean it was fan—they were fantastic, and I suggest anybody to learn more about hospice and what they do— because they’re fantastic. (11)  …the neurologist brought it up to me, but she brought it up in July, and, uh, the neurologist was great. She was—she truly was a great doctor, but there was—there was a couple of things. When I asked her—she always talked to my mom… The one thing was, when I asked her in July, “Where are we in the progression of the disease?” she said, “How does this help you? How does my answer help you?” (8)  .. in the end the doctor said, "You need to have a facility for him to go for, um, for hospice (9)  {Facility Director Name}, that was the director for memory care, she asked me if I would go to some hospice meetings, just to get a feel of what hospice was all about. And I am so glad I did that. I am so glad I did that. They helped out so much, and because it was getting to hard for memory care to take care of him. (11)  Very much so. Very much so because they—they could see a de—he was declining so bad. Speaking of family being onboard with hospice.  Hospice was, they were my salvation along with, um, two really good caregivers, you know, that I relied on. Um, we, you know, through Hospice, you know, we did some art therapy when we could. We did, um, a woman who played the cello or violin or something came in and played for her, but, um, uh, you know, I, I couldn’t have done it without Hospice. (13)  …the relationship that I had with the Hospice nurse. She was very, very responsive. Um, and the medical director of the Hospice. We used (Hospice Name}, um, Health Hospice. Um, the, the physician, uh, it was a woman doctor. Um. Who was head—so they all worked in conjunction with my mom’s primary care, but, um, you know, if the behavioral symptoms worsened they were right on it. Um, I think it was not having to jump through hoops. (13) *Speaking of what went well*  she started losing a tremendous amount of weight, that’s when they said to me, uh on the floor, “Do you wanna call in hospice?” … this is something kind of a key point, which you’re probably hearing a lot of; people don’t really know what hospice is. and they don’t know that it’s not necessarily—it doesn’t necessarily mean she’s gonna die in three weeks. , Mom was on two-two different rounds of hospice, so she was on hospice for a year. It’s like a six-month and a six-month. (14)  And what hospice meant, and what I was—when I was—when I found out about it, this facility had just started their own hospice. They just got a buncha money to start their own hospice in the facility, so that was kind of great because—it’s kinda folks who already work there, and it just meant that Mom didn’t have to be one of eight people who had to be washed and gotten ready for the day by one CNA. It meant that she had a—I uh—an aide who could, you know, um bathe her - in the bed, and could get—you know, take her time to get her up when—or she wasn’t too weak to get up, then, you know, feed her in-in the room. Um, and also—so it wasn’t a 24-hour hospice—- in the beginning. Um, it just kind of meant that you had more—a little bit more resources, a little more time. (14)  And what hospice was good in that point was that they didn’t let her uh—they said they wouldn’t have her be alone at all, so they had a hospice and aides sit with her—- 24/7, so that was really great. I don’t think it was all hospice. I think it was also some aides from the floor. So that was wonderful. They promised me that they would not leave her alone. So that was wo—that was wonderful. (14)  So we actually started hospice, which was very—I have a—a lot of respect for hospice. He actually was on hospice for nine months. (18)  hospice stayed with us that entire time. Um, he did have to get, um, re—I don't know if recertify is the right word— every three months. (18)  so though even though he got the care at the facility, but the hospice there—they came in twice a week with a caregiver who showered him and shaved him and, um, did his—you know, did his personal care. That, for me—for me, that's another thing that I, um—I feel very strongly about, that these people should be given the dignity and respect that their hair is combed every day - and that they brush their teeth and that they—you know, that they give them that respect. - and that they brush their teeth and that they—you know, that they give them that respect. So the hospice did that. So the hospice did that. (18)  We had a chaplain that came in every other week. We had, um, uh—[clears throat]—a music therapist that came to visit him every week. We had a nurse that was there. She was there to see him directly twice a week, but if she was in that facil—she was always in that facility every day almost— she would check on him anyway. So, for me, that extra level I felt was so helpful. And they also—of course, they sat down with me. They helped me. I was all on board with that this should be a natural process, that he should go—he should be at peace…And—and, of course, they helped me with that, but they also—oh, counseling. That's what—they had a social worker. They had a social worker for him and a social worker for me. And that helped me so that I could speak—he has four daughters. It helped me, um, working through that with them, um, and to understand the differences of—you know, we don't give him extra hydration at the end of life. and that type of thing. You know, let it be as natural. So hospice was—was, um—I would say that they did an amazing job. (18) *Speaking of facility with hospice*  And that helped me so that I could speak—he has four daughters. It helped me, um, working through that with them, um, and to understand the differences of—you know, we don't give him extra hydration at the end of life. We don't— You know. And—and that type of thing. You know, let it be as natural. So hospice was—was, um—I would say that they did an amazing job. (18)  Um, the hospice nurses were seeing her daily at that point, and she—the nurse that afternoon before this breathing difficulty began discussed the possibility of putting her on oxygen, um, because-uh, because her oxygen, etcetera, seemed low— but said, “At this point she’s comfortable. I don’t wanna put a cannula in her nose and make her uncomfortable”— “so let’s just, like, wait and see.” (20)  The hospice people were to a person just excellent. (20)  the best of part of Hospice was the things that the, the volunteers that came in and played a guitar for him cuz - he was a guitarist. And they sang. And the Reiki person came in and, um, uh, the, the pastor came in. and those were things that I felt he responded to. I mean I could tell that he was here and, maybe not right at the very end, but through those ten days all of that was good. (21)  hospice did everything right, and that was good, and they did what was necessary to get him the-you know, to get him the freedom and to get certain equipment and all of that. But it wasn’t the experience some people described of… So, I mean, it’s-it’s more (22)  he finally got into hospice mid-May. They came regularly. Um, he liked one of the hospice nurses. Every other hospice nurse he was very suspect of. (23)  Um, um other good things there, the hospice personnel were savvy enough to understand what to tell my mother, who’s the wife,— and what to tell me, the daughter, who’s more of the information-seeker. And they were very, very good about—the demeanor with me was far different than the demeanor with my mother; very comforting with her, very reassuring. “He’s not in pain.” He’s no”—that’s the other thing. He had no pain. It was absolutely amazing. He was in no pain. Discomfort from the catheter, but no pain whatsoever, so that was just really amazing. (23)  And hospice guided us, the care givers and my brother and sister and me …(24)  And I-I tend to think that there’s a difference in the quality of care between non-profit and for-profit. And we were very fortunate to have extremely compassionate—uh, it’s a team. They-they had a team approach. A social worker, a nurse, a physician. Um, someone specifically assigned to us. Uh, they-they really uh, set us up well. (24)  And we—hospice was really good in that they didn’t take offense that we had this team already, you know, who bathed mother and fed mother. (24)  I can’t say enough good about hospice. I am a champion of that. I can’t say enough (24)  I double-checked to make sure mother’s pulse and this and that. And-and I called the hospice nurse and um, um, she—to tell her she’d passed. And uh, she-she was very sweet. She gave us a little time uh— before [clears throat] before she came. And uh, uh, and then uh, you know, just-just full support. Even—and for a year after mother’s passing, you know, they would check in on us. (24)  where hospice was really helpful *[Laughing]* was um, um, just kind of helping us along with um, I remember it was very ha-ha—very helpful for me when uh, you know, mother would have dips, but then she would bounce back. And I was like how do you, you know, how do we proceed with this? And the hospice uh, nurse or social worker said well, you know, the dips, the highs and the lows, the dips and the bounce-backs will become uh, less dramatic. And uh, and she will bounce back, you know, less and less. Or when she bounces back, she’s not back up to par. And then she was right. It just was very gradual and uh, and you saw that the decline, you know, was occurring. (24)  yes, the hospice situation was a positive thing, and the fact that they were there when I needed them, you know, when I needed somebody to call. You know, “He just fell. What do I do?” A nurse would show up, and she would give—she would give me options. “You can do this. You can do that. What do you recommend?” or, you know, “What do you think?” And—and she was kinda there holding my hand, [laughter] you know, through—through all this, so, yes, my—my hospice pa—both with palliative care- and with the—the end of life, I—yeah, I—I had very good feelings, a very good experience, I would say, with—with hospice. (25)  They were wonderful. They were gracious. They were compassionate, but—and they loved {Name}. (27)  He enjoyed, you know, simple things. He enjoyed getting a bath. He loved having a bath, or shower, rather, from the—from uh the caregiver that came— from the hospice. (27)  Hospice actually got involved um in September—right as soon as uh Medicare stopped paying. We got hospice involved, uh because we wanted the extra help - and um they took over the medications, and um there was somebody there twice—at least twice a week, and there’s a—they’d come if you called them and said, you know, “We’ve got a problem.” They were very good with uh uh when the uh care center would call and have a question. They were very helpful. Uh, they worked well together with the care center. There was no animosity between them. Um, I was kinda concerned about that. And um, there—in fact, the care center was happy to have the-the extra help. (27)  when {Name} began to get some bedsores, uh and he had a bad back anyway, they uh ordered a mattress, and it was there in a day for him, an air mattress, a special—- mattress. Uh, any special thing, a special cushion for the wheelchair, they were really on top of things, and they had a chaplain that came once a week, and uh they would always r-report things to me at what they— saw and what uh—if I wasn’t there when they came, and uh we really—uh I can’t say enough good stuff about this hospice. It was wonderful. Uh, and when {Name} passed away, they-they came there and h-and uh called the um Neptune Society for us and just handled everything um while we went to make arrangements for the memorial service. So it’s a wonderful experience with this hospice. (27)  I was-I was happy for the transition to hospice- not that the visiting nurses weren’t doing a good job. But the transition went well, and I liked - um, the people I met, the-the chaplain. In fact, she and I have hit a friendship, and, um, she’s coming over later this week. Uh, we’re both pianists, so [laughs] that kinda - you know, enabled that. And, um, so, getting everything to happen quickly went very well. Um, the plan that the hospice organization has in place is, uh, obviously well-thought-out. Um, and, you know, that was by chance. You know, had he not been at the daycare, um—- wouldn’t have had that. Um, um, oh, the-the equipment company that they used they were really good. They were very helpful. Um, but that’s really all I can think of. (28)  But he had a girlfriend there, too, and she was really nice, and we still go over there and check on her, make sure she doesn’t need anything…And she still has his picture on her nightstand sayin’, “See this guy here? This was my boyfriend. He died.” And she came in to—while he was dying and h-held his hand, and hospice played music. They had a girl that sang church songs, and-and it was really nice. I think she played the violin or somethin’, but I’m not sure. Wow. M-my mind blanked that out, but I remember she sang, and everybody came along to his room to listen to the music. She was a music therapist, and it was very beautiful. And his girlfriend came in and held his hand, but when I go to see her, she shows me that picture and says, “This was my boyfriend. He died,” and this and that. And I’ll say, “I know. He was my dad,” and then she’s all happy, you know (29)  he was on hospice all that time. But, um, he was reevaluated by a-a good, um, uh, well he was more than a GP—he was a gerontology—- doctor too. And, uh, he said, you know, that definitely he needed, um—that he qualified to continue on hospice. Um, so at that point then, um, it worked out quite well. (30)  And, um, hospice was very—was very good, um, the nurse and the doctor and any time I-I called, you know. I would get an answer. (30)  **Sub theme: Negative hospice experiences**  I wasn’t pleased with the intake person. It—I really didn’t get any sense of, uh, compassion or sincerity. And, uh, just thanked her for the information she gave me, and-and I said, “Well, I’ll just see how this goes.” (7)  ..there was a mix-up at the doctor’s office. To make a long story short, uh, they (Hospice) didn’t come on Monday. On Tuesday afternoon, I called and said, you know, “I, um, kinda made this decision. It’s been very difficult, but where are we with it now?” You know, I—and so, um, you know, make a long story short, they didn’t know who to call, and it was just a mess. So, a-anyway, they ended up giving me a name of a difference hospice agency (7)  when the nurse came to see him on Thanksgiving, about, you know, he looked at the-the strings hanging down from the I had mentioned something about, you know, he looked at the-the strings hanging down from the overhead lights, and he thought they were a noose. I mean, it wasn't— anything, um, ex—you know, anything I was worried about. I was just relating to her how things had been going. And she suggested Haldol. And I didn't research it. (17)  when I talked with Hospice she just said to me, “You need to make that decision soon and it’s up—you know, we can’t help you with that. You have to make that decision.” Um, and she didn’t come out to talk with me. She did this over the phone and she said, “You let me know what your decision is and then I’ll come out.” (21)  I didn’t realize towards the very end that we had this odor and we kept him really clean. And my caregiver finally said, “{Wife’s name}, you know, it’s the bag. It’s the cath bag. That needs to be changed.” And I said, “I don’t have anymore.” So I went to the Hospice nurse and I said, “Could I get another one of those bags back because I’d like to change the bag because it’s, you know, it’s, because he’s dying his kidneys are”— you know, it’s not. She said, “What do you need that for he’s dying?” So I said, “Well I want those back,” and I said, “I will give whatever is extra back,”—I had bought them myself. I will give whatever extra is back to you, but my husband deserves—and it’s not, you know, it, it smells. I mean you know— you, you don’t want that. You know, I mean at the very end when you know your husband’s dying or your wife you want the very best for them. (21)  didn’t tell you the worst part of—it just blanked out. They knew he was dying, and, um, when he passed, it was a quarter to 4:00 in the morning, and I’m there. I don’t wanna leave him alone. You know, I just don’t wanna— leave him alone. So I-I-I ring the call button, and nobody comes, and nobody comes, and nobody comes. So I go to the nurses’ station. Nobody’s there. I find an aide, and I say, “Can you please—where’s the nurse?” “Oh, she’s not on the floor.” “I need the nurse to come.” So she comes, and she says, “Now I have to get another nurse to-you know, to, um, kinda certify,” whatever the word is, “to-to pronounce him dead” “’cuz I’m not”—he—so the other nurse comes. And then she says, “You-you know that-uh, we know that you’re gonna do brain donation,” and I had been told that what that means is that his body needs to be transported to the lab within-uh, within, uh, 6 to 12 hours or something of the time he passes. So the second nurse says to me, “Well, we have to wrap his head in-in-uh, in ice right now.” Um, and, um, I was sort of—I said, “Okay, but we need to come back.” “Well, now we need to do—you know, we need to take him outta there.” I said, “My son is on his way here. You’re not doing anything.” And the whole—it was terrible. I mean, it was really upsetting. (22)  And hospice-hospice came by every day starting that previous Friday, but hospice wasn’t there longer than an hour max. Uh, I think um my a—my idea of hospice would have been for someone to be there more. Maybe they were too busy. Maybe they had too many other cases. I don’t know, but—or maybe this is just their practice, but if the person i—the-the hospice patient is in their own home, the hospice nurses only come once a day. If the—- hospice patient, obviously, is in a hospice facility, you’re gonna get more service. Anyway, that’s kind of an interesting thing, and-and understanding that, I think, would be a-a—something for people to know right off the bat. I don’t—- think people understand that. (23)  Um, my mother really waited to call hospice. I don’t think she was ready for this to happen. I kept updating her of, you know, “And now this is the next phase, and now we have this next phase,” because as soon as hospice came in, I wanted to know all there is to know about what to expect, because—- I needed to manage the situation, and I needed to manage her. And I’d—- separate those two out. Um, I did a lotta research on the Internet. There’s a lotta great information on hospice, so kudos-kudos to hospice for having lots and lots of great information. Um, um but yeah. Um, and hospice didn’t ultimately come until just after midnight, so technically when hospice comes and calls time of death, that’s when they’re there. He passed on the 6th. The time of death was really the 7th of June. Still, we’re gonna call it the 6th. (23)  But uh, hospice uh, the negative on hospice is that I asked around in [City}, Texas, which-which—what are—who are the top three? I kept hearing that the top one was this particular company. They were a for-profit uh, hospice outfit. I signed up with them. They uh, the very first day just immediately fell apart, as far as not-not being there on time, not-not—or-or—it was something really dramatic that I just thought well, we’re not doing that. And uh, so I uh, ended that relationship before 24 hours were out, and then hired a uh, or secured a-a non-profit hospice. And I-I tend to think that there’s a difference in the quality of care between non-profit and for-profit. (24)  And supposedly, that-that was the group that would come in at the end, the last few, um, wh-when he was really in the dying stage, and be there 24 hours. And so, I chose that. It turns out that I was very, very unhappy with the kind of, um, response that they had. Uh, just a quick res—you know, kind of an—a-a-an example. Um, on Friday, um, he was in the nursing home. And we met and got this—th-the great sales pitch of, “We’re so good, and we’re so great because we’re so big.” Um, and, um, we signed the paperwork. The nur—the nurse and the doctor did not come to visit and do the initial eval until Monday night at 7:00, when he was really exhausted by that time. And at the end of that, um, that-that eval, they brought me back to their little office they were, uh, using. And they said they wanted him just left in his bed. Well, over the weekend, the-the caregivers had been, you know, putting him in the wheelchair and bringing him out and putting him in the—you know, bringing him to the dining room and putting him into the-the lounge chair where all the familiar and where people were all around him. And I started crying. I, uh, you know, I know that means. Let’s just close the door and let him die. You know? (26)  There were many incidents like that where, um, he had a-a huge, huge, um, blister on his heel. It took me two days finally actually having a fit, crying and everything, on the phone, before they would—they sent somebody out to take a look at it. Um, and, oh, the reason they—no. Somebody had come out to-to look at it that night because I threatened to call the state… and he said he needed some kind of a—like a boot, or something, to protect that heel, cuz he was bedridden by this time. And, um, it took them—uh, i-i—that was on a-a Sunday night, I believe. And I couldn’t get them to respond. And finally, they sent out what they needed to send out. It was just—it was one incident like that after another. (26).  the-the-the last incident was—the, uh, the last four days. So, he died on Thursday. So, Monday night, the director of the summer house, the nursing director, who was just a wonderful, uh, person—her name was {Name}. And {Name} finally called {Name of Palliative Care Company}. She’s the one who convinced me to take {Name of Palliative Care Company}. She finally called and she said, “You need to bring somebody in here. This is now the time to do the 24-hour.” And finally, they brought somebody in that-that, uh, Monday. Um, so, I had 24-hour care. People came and went, and came and went. When they were very—the nurses, themselves, were very good. (26) *Speaking of nursing home director advocating for more hospice services*  And the nurse said to us at about 7:00, “Well, I have to leave at 8:00 and, um, and there’s nobody here, nobody to come until, um, the, um—until 3:00.” And I was like gasping. I wa—and-and she says, “Well, I’ll show you how to suction him.” And so, that was, uh, it was just—it was unbelievable. He died at 9:16 that morning. Um, she did not leave. She ended up staying, I think because she knew he was dying. (26)  So, in the morning, when we got that-that, um, the nurse said, you know, “I’ve gotta leave at 8:00,” um, and, um, and so, she, um—I-I was sitting by his side, and she was across the room. And she said to me, “You can tell him he can go now.” And I was sitting there thinking of how—I mean, I was pretty emotional at that point, anyway. And thinking, “How did—how do I say that?” And I was tryin’ to think it out and word it out in my head. And then she called out, “You can go now,” to him. And it was so upsetting to me. And I also wonder, because of that incident, yes, he was on morphine. Did she up the morphine that morning? Did she hasten it? And, you know, it’s those things that you don’t know.(26)  And that was—it was stunning to me. The whole experience was stunning because I had always heard such good things about hospice. And I had such trust going in. And it was, um, not, you know, validated through our experience. And, um, you know, maybe I’m a little bit of a perfectionist. I guess I am, but, you know, when there’s—there has to be a trust built in. (26)  So, you know, the-the time I most needed somebody to be there with me, it didn’t happen(28)  Well, we can’t do all the paperwork and the transfer to get you back onto hospice again, um, and, uh, you know, align everything, the equipment, until the next day.” So, uh, it was Wednesday when she told us this, and I was like—- uh, got my hopes up and then have to wait again. [Laughs] (28)  With one group, um, the first group we had, um, he start—my dad like failed like really quick where he wasn’t gonna eat or drink anything. He looked horrible. Just super stiff. And, uh, his face was all mask like and he was just kinda out of it. And, um, I told him, I said, you know, “Don’t feed him. Just, you know, let him go. I don’t want him havin’ any food or fluids or tube feeding or anything like that—,” you know? And, um, he was dy—actively dying, and they were medicating him to keep him calms. Well, those idiots gave him a PRN pain medicine or whatever they were giving him to keep him sedated and clam. They ordered it PRN. So—- it had been two days without food or water, and, usually, it’s about three days, and-and they fall asleep and die. Well, I go in there, and, all of a sudden, my dad’s sittin’ up in the chair, and somebody’s feeding him. I’m like, “Are you frickin’ kidding me?” Oh, he’s havin’ a flight into health.” I got, “No, he’s not, and I look, and they didn’t give him his pain meds cuz it was PRN.” I’m like, “Are you frickin’ kidding me?” Oh, he’s havin’ a flight into health.” I got, “No, he’s not, and I look, and they didn’t give him his pain meds cuz it was PRN.” And they thought he was fine, so they took him off the stuff that was dopin’ up to help him pass peacefully. (29)  **Sub theme: Early Hospice Discussions**  hospice, uh, came up early and often, and we got—I think she was in hospice for nine months maybe (2)  So we actually started hospice, which was very—I have a—a lot of respect for hospice. He actually was on hospice for nine months. So because of these incidents, what I talked about in the beginning, where he had several very aggressive modes (18)  about a year—just over a year before she died, we made the decision to-um, to enter hospice—- or to have her enter hospice.(20)  we—in the course of a conversation about that, you know, regular care conference, the social worker asked if we had considered hospice care for her. And at that point, we-we hadn’t. She was ambulatory, and-and we hadn’t—we didn’t have much of a handle on what was going on with her, um— we spoke then with her primary caregiver, and she felt that-um, that would certainly—that-that [{Mother in Law} would not qualify for hospice care at that time. And then after we moved her into the memory care facility, in a care conference there, a nursing— supervisor not associated with her care, she hadn’t really even met her, um, raised the question again: Had we considered hospice? And so, once again we spoke with her physician, and the physician told us at that time probably not yet, but we should talk about it in another six months. And then at that six-month mark, the doctor, uh, in a regular visit to her, um, and talking with us afterwards, said she-she felt that it would be appropriate for us to— get her hospice at that time…. we wanted {Mother in Law} to be able to have the benefits of hospice care— as early as it was appropriate. So, we did immediately enroll her in hospice then. (20)  when he had fractured his hip a year before, I had started interviewing hospice groups (26)  …toward the end of that three-, four-month period, um, she mentioned something about hospice. . And I said, well, um, I didn’t really think we were ready for that. I mean, he was still up and around. (28)  **Sub theme: Hospice Discussions (Negative)-**  I'd asked them about hospice, and none of them even recommended hospice. Um, there was really no discussion of what the end of life was gonna be like for her (3)  it just all happened so fast, and it wasn't like they were bringing those things up soon enough. (3)  we did not get on hospice when we should have, and I didn't know any better. (15)  Nobody mentioned the word hospice. Nobody ever said it would be a good idea to look into that now too. Um, I didn't think of it. I should have, but, you know—and of course, we didn't know how end his near was. He was ‑ he was only two weeks in the home before he passed away. (15)  Nope. Nor did the woman ‑ nor did the woman who ran the ‑ the, uh—the adult care home say anything about that. It was 24 hours before he died that she said, "He's really not doing well." "He should be on morphine. You need to contact hospice." Now, we had had—I think we had a physical therapist come out and try to get him to stand up there and talked about using a Hoyer lift, and the woman, uh, who ran the ‑ the adult home said, "I don't think you're gonna get him to stand up. We've been trying and trying." (15*) Speaking of no hospice discussion until last 24 hours of life in adult care home*  Well, what it came down to was that was the way we could get him morphine. And I had to literally drive like the wind to get to the pharmacy as fast as I could to get him the morphine, but we couldn't do that until ‑ until hospice was contacted and said, "Okay. Get him the morphine." So it was ‑ it was a madhouse. I was calling, calling, calling, and trying to get somebody to okay this fast. So then we did have a hospice worker come to the home, but, um, you know, what was she able to do for us? You know, it was just a preliminary look, and so we never really got any help. (15)  **Hospice Discussions (positive)+**  but it seemed like a long time before he met those criteria of hospice. And-and that would be—um, the decision to withdraw food was-was the—a-according to the, um, ce—the center where we-we were at, that was the-the cue that you could call hospice. (5)  we’ve been under hospice for-um, for a year, and that decision was made by the nursing staff and the, uh, physician that would come in and say, “Well, she needs a little additional care.” (12)  …one of the times she had fallen, um, I ended up taking her to the hospital. I was the one that had to bring up is she a candidate for Hospice. Um. (13)  I made a call to home health, and I said, "She's—this is what's happening. He's getting weaker, and now the aide can't get him up. I'm a little worried about keeping him at home." And they suggested hospice was the next step. And within two days, or the next day, they were down for me to look at the papers, sign the papers. And the next day, uh, we had all the equipment we needed to care for him at home. (16)  I was actually thinking of possibility of a nursing home. I really thought we had a much longer time—- for us, and I was looking at the-the pr—what I thought was the probability of a nursing home. But because he suddenly, um, was so weak that the-the aide couldn't care for him, um, they're the ones who suggested to come in with the equipment. And it really was a blessing that they did that. (17)  The same neurologist, about six months out, right before Christmas, told me that he had—he—it was now time to um, to call hospice. And uh, he told me that uh, he-he-he was—he had done all he could. And uh, that it was now time for her to enter into hospice, and that that would be a different relationship. And uh, which, of course, it was. And uh, and he was accurate. He said he thought it would be about six months, and he was-was very uh, that was pretty-pretty spot-on. And uh, so we worked with hospice. Um, uh, uh, we worked with hospice um, she was always, again, at her home. (24)  when I talked to, um, the gal at the-the daycare, which he went to one day a week by that point, and then they had, um, a hospice nurse come in there once in a while because she was visiting one of her clients… And when I was talking to the gal at the center, she mentioned that whenever I was ready for hospice, this gal was very interested in taking on my husband because she just loved him… so, at that point, it was like something clicked, and I said, “Okay, let’s do it.” And I wish we had done that m-much before. It would’ve been so much more helpful. But I didn’t know. Nobody gave us full information. I knew hospice was not for just, you know, in their last days or weeks. I knew that they could do more than that. My mother had been on hospice her last six months or whatever. Um, but I didn’t understand the scope of what they could do for us. (28)  **Difficulty Getting in**  we were thinking that we would take her there; um, they would, you know, hopefully be able to send her home and get us attached to some hospice…- 'cause we'd been trying for several weeks to get attached to a hospice (3)  I-I would have, um, found a way to really push and get hospice in there sooner-- um, because I just think that that would have made a huge difference I would have gotten ho—the hospice and started working on end of life— (3)  when they finally called them, I-I mean, it was like, oh, my gosh. Finally. You know, that’s a horrible thing to say, but finally, we get hospice… Um, but it seemed like a long time before he met those criteria of hospice. (5)  they saw the writing on the wall, and I think they knew that he was destined for hospice, but because at that moment, which was M—early May, um h-he didn’t meet all of the criteria at that time to be— admitted. It wasn’t another two weeks until he finally got admitted, so mid-May. He was in hospice maybe two, three weeks, and he passed. (23)  So, finally they said—she said, “Have you thought about hospice?” And I said I thought it was too early. Well, she helped me, and the-the first hospice said, no, he’s not—“We won’t take him,” essentially. They didn’t think that he met the guidelines. And so, there was another hospice that said yes. So, he was in hospice for, I would say, about six months before he died. And the difference that that made was he had more freedom. I mean, the—he was able to—if we had more space, then it was less-less agitated. (21)  ..the one thing that I would offer is, because of the rules that hospice has to follow, my father didn’t fit in the box of their rules—- until two weeks after they were first called. I mean, we called them in—well, the doctor called them in—um, oh, that’s the other thing. We—uh, the doctor called them in, and they—and h—the uh lovely woman, I think her name’s {Name} 24:35, came and she said, “Oh, yeah, yeah, yeah. It’s just a—it’s not today. It’s just a matter of time.” Well, I could have used you today. You know, I coulda used that little extra support. Um, again, it didn’t happen. It didn’t happen for a reason. Whatever that reason is, I don’t know. I think if-if hospice sees the writing on the wall, is two weeks really gonna matter? I mean, seriously, is two weeks really gonna matter um when you see folks kind of struggling? (23)  So they came and did an evaluation, and that first time, he was—he was too functional. They said, “No, uh, I don’t think he really needs it,” so he didn’t. And then he had declined. Remember, I mentioned after a certain amount of time—- he had really kinda dropped off. They came again, and at this point, I think I was feeding him. I don’t remember exactly, but he—he had just declined a lot in terms of what he could do for himself. (25) *Speaking of hospice eligibility*  my son is a teacher. He works with another teacher who has friends that had a hospice that they had uh started a few years ago here in {City}. And we got a hold of them, and the doctor in charge of the hospice came to spend an hour with us one evening and explained the whole thing. We thought you had to have at least—uh only six months to live, and uh that’s really not the case for hospice, we have learned since then. And he was extremely helpful, informative; a wonderful man. And-we said, you know, if we can get {Name} into a hospice, this is what we—this is the one we want. And um they helped those last months, um because there’s never enough staff um - in the nursing homes to work with the people that really need more care. (27)  **Sub theme: Family impacting Hospice Delivery**  Dad had stopped eating and drinking, she was like, "Well, can't we put him on fluids? It just seems cruel." I said, "Does he look like he's suffering?" "Well, no." It's only to make yourself feel better and he had signed—- years before what his request was. I said, "Please don't do this to him." I said, "He is not going to survive." (9)  I don't feel that from his PCP perspective, when she would take him in and explain things to the doctor, like that he was declining, that she was—my mother, that is—was offered, um, any type of additional care—- for perhaps like end-stage—the end-stage Lewy body dementia. Like hospice or palliative care support. (1) *Speaking of having to take over caregiving from Mom- who was previously primary caregiver*  **Process- no Eating and Drinking**  And then when we—when-when we finally came to the decision with hospice to withdraw food—they told me it would be about 10 to 14 days, but it ended up being more like 3 months. It was awful. (5)  It was very surreal, actually. They—the, uhm, the, uh, hospice team was nice. It was a little shocking because we didn’t realize that my dad wasn’t gonna like be eating or drinking there. (19)  **Lack of continuity across hospices**  the way they worked it in {Florida} County, or at least in Florida, this part of Florida, the hospice had two different groups based on county cuz we’re now in {Florida} County. So we didn’t have our old hospice group that he had had for palliative care. We now have a group, but they were—they were still the same organization. (25) |
| --- | --- |
| Hospital Palliative Care | **Positive Palliative care experiences**  The palliative care at the hospital was very, very receptive to needs, and then at that point the staff really was on board with, um, helping, you know, keep him as comfortable as possible, assisting with our emotional support needs and stuff. So there was a big difference then. (1)  the hospice care that they offered here at the hospital, uhm, was amazing, the hospice that my dad was. We had to make the decision (19)  -the-the hospice, they were just—and I mean I-I’ve never had any experience with hospice, so I didn’t know, you know, but I did— when we were looking into facilities for him, [distorted audio 17:02] looking specifically at places that could bring hospice in - or he wouldn’t have to transfer again because, uhm, we were told that, you know, the—I guess the, uh, they’d get routines, you know, and it messed the routine up or to change environments could— could not be good, you know (19) |
| Medication use | **Medications used during course**  She was on that, losartan, and—it was like those were the only two medications she was on, the galantamine and the losartan. (7)  ..managing his, uh, disorder, with, um, clonazepam and melatonin—the suggestion of melatonin, um, was wonderful, you know? That, in itself, was great, that we had that, but, um, I'm not sure that, as far as relating to Lewy bodies, that anything else went as well. (17)  And I will tell you that, um, throughout all this whole—this nine to ten years of this, there was a lot of experimentation with medications. (18)  And you can't give—there's a—a—you can't give Alzheimer's medication to Lewy body dementia people because we did have, um—he went—at one point, they put him on Namenda. He had a suicidal. he—because he was gonna kill himself because of this Namenda, and he'd only been on it for 24 hours. (18)  I think we finally got to where the medication—he was on very little medication at all. (18)  Um, I uh—my father was diagnosed with Lewy body dementia maybe about five years ago, um and he was put on a drug, rivastigmine; wonderful drug, love it—um, along with, you know—he was on metformin. He had Type Two diabetes. He was givli—giving himself insulin twice a day; sometimes uh towards the last couple of months, three times a day. (22)  …the neurologist, every step of the way with the medications that he prescribed um, he—in the end, uh, um, she had—or for a great part of those four years, she was on uh, a medication for Parkinson’s uh, uh el dopa—I don’t know what I’ll remember it. But-but medications for Parkinson’s and then medications, I know she had the uh, Exelon patch. Um, um, and uh, and then she had Tylenol for the uh, the osteoarthritis. Then um, um, then she was all of a sudden given an antidepressant when it was appropriate. When she was becoming so fearful. And uh, and then she also had another drug. Sera—not—no, Serafil. Serafil for the uh— Serafil worked effectively for her hallucinations. They went away. (24)  So, um, I mean he was taking Aricept, and then we changed him to Exelon. But aside from that, there wasn’t a whole lot else that he was taking. So, anyhow, I—I was, you know, was at that point where I’m thinking maybe Sinemet would be a good idea, um, but it never quite really happened. (25)  But we were not on any medications, and I really think that had a lot to do with it. Um, we did as much as we could through natural means—- both, um, diet and supplements. And, honestly, the one supplement that I know made a huge, huge difference is-um, is called Vitaline or Vitalin brand CoQ10. And it was used specifically in Parkinson’s research. That exact medica—uh, supplement was used in Parkinson’s research and was proven to slow down the, uh, progression of the Parkinson’s movements by five years. And I can’t help but think that that was hugely responsible for him doing as well as he did. (28)  Um, way early in the process, you know, one of the first things we noticed was the tremor in the left hand and, um, that he didn’t swing his arm right when he walked. But he was also starting to have trouble with swallowing, and he was choking, um, on his food before we really even knew what was going on. And once he started on that supplement, that choking went away 100 percent. And his tremor never got worse, and, in fact, often I thought, “You know, it doesn’t seem as bad as it used to be.” Um, so I know that that played a big role, and that’s one thing I would not trade. It-it’s expensive. It’s $150.00 a month. Um, it’s a chewable tablet, and he loved it. He-he—that was like candy to him. *[Laughter]* There’s a couple of different flavors. Um, so, you know, that-that is definitely something for anyone with Parkinson’s or Lewy body… And he was even able to cut back to two a day instead of three, which made it last longer than a month, so it wasn’t quite- $150.00 a month, um, after the first few months. (28)  I got him on supplements—Lexapro for not so much depression, but for irritability -associated with the periods of confusion that were frustrated to him, and it worked very well. Got him on a ton a—I spent like $250.00 on supplements for him that were all for the brain and research tested and everything. And [inaudible 06:14] and the Exelon. Then I had him MRI’d to make sure it wasn’t like normal pressure, hydrocephalus, or somethin’ else goin’ on. It wasn’t. His brain was pretty good, actually. (29)  You know, all the supplements I bought him, the, um, the Aricept and the Namenda, and then I got him on Symmetrel as he started to get, you know, decreased arm swing and movement. I have a sub-specialty in neurology. And, um, he’s had like decreased arm swing, and I noticed he’s gettin’ a little more stiffer. Then I added Symme—Symmetrel. Then I added a touch of Azilect for neural protection. Later on, I got rid of that cuz he was getting those hallucinations at night. Here’s another complex thing: My dad also had narcolepsy and REM sleep— sleep behavioral disorder… I had him on the baby aspirin as well and all the different herbs that were—are proven to help decrease inflammation in the brain and the body and neural protection. (29)  we put him on Luecntis and we had to-to regulate that, you know. (30) *Speaking of constipation*  There was only one drug that uh, good god, we just—it stunned us. It was gabapentin, which I hadn’t- hadn’t been afraid of, because my pups had had it for other reasons. I mean, you know, but we—I can’t remember why, but he—the neurologist said well, start her this. And mother went flatline. I mean emotionally-wise, emotion-wise. I mean she—her face—she just—it just was stunning. And uh, she lost all affect. And uh, I called him, and he said, “Get—no, stop it, stop it.” And uh, so we moved another way. (24)  **Medications for hallucinations specifically**  uhm, cuz he was on a different, uhm, anti-hallucinogenic that didn’t work well. It was kinda—made him lethargic, so he didn’t take it. So they put him on the Nuplazid and, uhm, really, it was like a week later, uhm, my dad had, uh, physical therapy comin’ into the home, although he-he—like I said, he was mobile. He was kinda getting—losing his balance a little bit, but like he wouldn’t wanna use his walker. He used a cane. (19)  It seemed like they were havin’ trouble controlling his potassium. I don’t know if that was coming from his cancer or if it was coming from the meds he was taking for Parkinson’s. He was taking the, uhm, carbidopa—- and then, uhm, he was, ah, I—then he started takin’ the Nuplazid and they really don’t have a lot of information on that—- cuz it’s a new drug, newly approved, so that’s what the neurologist said. She-she couldn’t say if i-it, you know, some things were coming from that drug or not. They just couldn’t be sure. (19)  She had—at-at a certain point, developed auditory and uh, visual hallucinations. And he-he—wow, he just really wonderfully controlled that with a medication. (24) *Speaking of neurologist*  uhm, cuz he was on a different, uhm, anti-hallucinogenic that didn’t work well. It was kinda—made him lethargic, so he didn’t take it. So they put him on the Nuplazid… (18)  **Conservative use of Medications**  I think we finally got to where the medication—he was on very little medication at all. And, um, summer came. (18)  his Parkinsonism was getting worse. And the neurologist was kind of letting me—he was—he was very conservative with respect to medications. Which kind of fit our philosophy, and my husband was has—was always happy with that. You know, he didn’t wanna take anything more than he absolutely had to. (25)  **Medications around end of life**  they were doing diazepam, morphine, uh, rivastigmine—- and that was a patch. And I think we stopped that partway through. And then, uh, they tried the olanzapine. I-I don’t think that really lasted very long. (8)  We had established care priorities of minimizing physical and emotional distress. And that meant sometimes that she was medicated to the point where she was sleeping more than maybe was ideal. But if she was awake and crying all the time, that wasn’t [laughs] a good alternative. And so, they worked well with us, I think, to try to assess where that sweet spot was and try to keep— her there. (20)  **Use of Pain medications**  I think they used morphine (5)  And the morphine, the liquid morphine, and all these liquids that they’re administering through the mouth had no way—my mom ha-had no way of swallowing it (8)  … as far as I know, he was on morphine. I don't know anything else, but that's all I think he was on. 'Cause at that point—I mean, he had had all these medicines, and they took him off the medications he had been on in the hospital. And I think he was just on morphine at that point.(4)  He, um, was on morphine, but they—Well, my brother was allergic to morphine, but they gave him such low dosages, that they would just squirt it in his mouth - that, um, it helped. And then they could give him more, you know, like every half-hour or whatever… (11)  It was clearly not intentional, but—but nevertheless, it kept happening. So like one of the reasons it happened was this stupid thing where they switched pharmacies, and the f—the shape and the size of the pill changed, so they thought it was, you know, a different level than it was - cuz it matched the other—you know, like silly things like that… so that’s why she kept being in so much pain, and that was, you know, excruciating, obviously, to watch. (2)  And during those five days, they kept him, you know, very sedated. And, um, was- he was very comfortable. He only opened his eyes one time during that time and it was really literally hours before he died. (9)  but the doctor still called us, um, that morning to get permission to administer the-the morphine at a minimal-minimum dose. Uh, and, uh, before it was—uh, this is probably another level that she’d reached that the end of life was probably approaching, and, uh, she probably would, uh, pass away within the next several weeks to a month, so we’re near the end. So, again, this is the kind of thing we were expecting to hear, and we went forth with the, uh, administration of the morphine. (12)  pain from those things on top of the lewy body dementia and trying to make them comfortable and, um, towards the end we really, um, relied on the morphine and, you know, it was my goal that she be comfortable. (13)  I think that it was, again, not-not really understanding um the morphine situation, like whe-when hospice can give morphine. So she stopped eating and drinking completely. Um, and then she just kind of—when—just was sleeping most of the time. Um, and-and then they wanted to reduce her pain because the body started breaking down, and they’d give her some morphine, but initially—I remember this. Initially, they gave her morphine, and she um uh uh—she had the ga—a gag reflex to it for a whi—quite a while, so it wasn’t—it wasn’t agreeing with her. So I was like, do we stop it, right? That was also a point of con—kind of confusion for me. Do we—do we stop the morphine—- because she’s gagging? It just doesn’t seem like a-a pleasant end of a life right now. Um, so we did… (14)  Then—she-she was-um, she was only ever given, um, an injection once, and that—and I had to go and say, “She seems”— - “to be in a bit of distress.” And I don’t know whe-I don’t know whether it was a sedative, um, or-or-or a pain relief— - morphine-type pain relief. Um, so, um— once. They-they administered it once, and I don’t-um, I don’t even know what it was they actually gave to her. (16)  Like he-he’d [distorted audio 18:00] pain medication, so they gave him morphine and they gave him, uh, Xanax or, uhm, lorazepam I think (19)  he was given, um, morphine and other things to make him comfortable. (22)  And uh, and for the first time the care giver—or any of us, the first time the care giver had to get some morphine to relax her throat. They had said that would help, and it did. (24)  remember that—let’s see, so it was definitely three, four days before they had told me to—that it would be okay to give her a small amount—a certain amount of morphine every hour. , I guess, OCD or just crazy about if you tell me a schedule, I’m on it. And I was setting my alarm and just every hour making sure she had her morphine. And it was a very little amount, but um—and I was okay with that, by the way. You know, I-I was okay. Um, I knew that uh, um, that ultimately it was about her um, her uh, peacefulness, her peaceful passing. (24)  It was just the, um—the {Florida} County, um, group. And so they started him, and I know this—this is a question that comes later. They started him on, um, a very—a low dose of morphine. Uh, sublingual, and at that point, he just became totally, totally unresponsive. Um, but—oh, but what I was saying is when he—before we had done that, he was still—he was sitting up, and I handed him a potato chip, and the first potato chip, he struggled and struggled cuz he loved potato chips. Struggled, and it got it to his mouth, and he was able to eat it. Gave him a second potato chip, and he—he couldn’t even get it to his mouth. I mean he had deteriorated—- that—that badly…., he was totally unresponsive, so for that, I—I don’t even know how many days it was, but it—it had to be a good week. (25)  No, no, I brought it up, and I asked that hospice come in so that he gets extra TLC and that he gets, uh, doped up a little bit so that he can make his transition peacefully. (29)  And the last, uh, two days or so, he would—he would draw for air. And so— uh, we had him on, uh, morphine, liquid morphine. And, uh, because you couldn’t really tell that he had any pain at any place in this. Uh, you know, well I would say since he got home, you know. You couldn’t, but, uh, uh, then we just kept doing that. And, uh, just had to just kinda let him absorb it, he couldn’t really put it in his mouth much. (30)  **Needing multiple medications for comfort**  They, um, started him on initially, um, morphine IV every two hours as needed. And then that wasn't really keeping him comfortable, so about—I'd say about 12 hours after we made that decision, they started him on a morphine drip. And then, um, they, augmented that with, um, as needed Ativan IV Robinul, and I think he received a couple of doses of IV Haldol, but I think that was just two doses… once they started the drip, he became more comfortable. Prior to that, it was a little rough, but they addressed it quickly. (1)  So he got his Haldol, and I can't remember what he got for his constipation. Um, I don't know if it was—maybe it was a Fleet enema that-that we gave him…., um, and he was groaning and moaning, and he was in terrible pain. I could tell he was in pain. He didn't know what was happening. Um, called the hospice nurse, and she was giving me instructions on how much of his morphine and lorazepam that he was on—- to relieve that. (16)  Like he-he’d [distorted audio 18:00] pain medication, so they gave him morphine—and they gave him, uh, Xanax or, uhm, lorazepam I think. Uhm, but he seemed comfortable and he, you know (19)  We had a great deal of difficulty getting her pain under control and put her on a series of, um, escalating-strength narcotics from the—uh, from, um, Vicodin to Percocet to, um, a morphine drug. And finally she got relief in transitional care at 36 milligrams a day of Dilaudid. (20)  he was given, um, morphine and other things to make him comfortable. (22)  **Use of antipsychotics**  I think he received a couple of doses of IV Haldol, but I think that was just two doses…. Not that I'm aware of. No. (1) *When asked if Haldol caused problems.*  Then, in a rehab facility, they administered Haldol… We had no idea this would’ve been a problem… when they administered the Haldol that he fell into sort of a—I wouldn’t say comatose state, but he was uh, retreated quite substantially, and he never really fully recovered from that. And so, he-he-he did come back a little, and he was, you know, he was communicative again. But uh, you know, he sorta just went through, then, a steady decline. (10)  we had some, um, medications that we were giving him. Uh, when the nurse came to see him on Thanksgiving, I had mentioned something about, you know, he looked at the-the strings hanging down from the overhead lights, and he thought they were a noose. I mean, it wasn't— anything, um, ex—you know, anything I was worried about. I was just relating to her how things had been going. And she suggested Haldol. And I didn't research it. (17) *Speaking of Hospice suggesting Haldol for hallucination*  I would say within a half hour—and this is about two, two and a half hours since he had his Haldol—he had a neuroleptic malignant—episode. He was suddenly sitting upright. He was completely—every m-muscle in his body was clenched. His mouth was clenched. It was opening and closing, opening and closing. His tongue was - was, uh, thrusting out. He almost bit his tongue off at one point, um, and he was groaning and moaning, and he was in terrible pain. I could tell he was in pain. He didn't know what was happening. Um, called the hospice nurse, and she was giving me instructions on how much of his morphine and lorazepam that he was on to relieve that. His temperature skyrocketed. At one point, I got an axillary temp of 104. Um so all his covers came off. I was putting cold compresses on him. I turned the fan on a-above him, trying to get his temp down. So, basically, I was left to manage all this by myself. And I am an EMT, but I'm also, you know, the wife and the caregiver, and, um, it was al—it—I-I can't imagine somebody else going through that alone. (17)  And it wasn't any time to bring in help. I mean, it was so sudden. Everything that was happening was so sudden that it just—I just needed to react immediately to it. Um, and, from there, his kidneys shut down, and he wa—he was gone by Tuesday morning. And I truly believe that it was the Haldol that hastened his death., and my—the inexperience of the nurse who suggested it, the inexperience of the provider who okayed it— (17)  he was bedbound, I think the first or the second was maybe a Thursday or Friday—by about Friday night I had to start giving him Haldol um from the hospice comfort pack. And that started once every six hour—once every eight hours. Then it went to six hours. Then it was almost once every four hours, um until about s—Monday, M-Mon—uh Sunday my mother had ordered a hospital bed because he kept moving around the bed, and even though, believe it or not, they were still in the same bed, um he was moving around so much and he would hit her. (23)  Um, they—the staff, they had to—{Name} uh—when he was combative, they did have to give him medications to calm him down. And um—and that was probably a good thing. I mean, I hate to see so many medications given, but uh it’s better than him hurting somebody else or himself, so. (27)  **Use of anxiety medicine**  I knew her, I knew that it was her anxiety. And it was only the anti-anxiety that really helped her calm down and feel better. (3)  So, you know, there was the regular medications, and then there was as needed. And then, at the end, it got to be—I mean, we could request the anti-anxi—anxiety, like, every hour, if we needed to. So that helped. That helped. (3)  **Question if patient needs medication(s)**  And I’ve learned that when the patient gets to that point where they stop eating and stop drinking, and your body is starting to shut down, they don’t know they’re hungry, and they—- don’t know they’re thirsty. Those things shut off, and they’re not in pain for—well, a certain time, I believe, they’re not in pain. And-and hospice, though—one of the things to help on the pain—well, if she had pain, of course, is they gave her morphine…. I don’t know how to answer that. Um, I can’t say she needed it, but they said she needed it. How do you know? …I don’t know- if she needed it, but they felt she did. (6) *When asked if wife needed morphine*  And he was also on, um, I think it was called, uh—I don’t know if I’m pronouncing this right, uh, Brazepam.  (11) *In addition to morphine*  **Use of Anesthesia**  And he certainly got worse after each procedure. And the anesthetic, I think, really screwed him up. I-I don't know if people are aware. I certainly wasn't, 'til I sort of—you know, that, uh, anesthe—when people have surgery and anesthesia, with those conditions, it's horri— deteriorates things further (4)  he had cataract surgery the next day and then that’s when the doctor had told us with the anesthesia and, you know, all of this, he didn’t know if my dad would ever really recover and he didn’t. (19)  So when {Husband} came out of the, um—out of the anesthesia, I really saw no difference in him. (25) *Speaking of spinal anesthesia during surgery*  **Medication errors**  one of the bad things that happened, um, repeatedly for my mom is that they kept messing up the morphine level, so Um, just the—the whole idea of, uh—what do you call it?—the coordination of care there were a couple of different errors that happened, you know, well-meaning people. (2)  … no one wanted to do harm to her. It was clearly not intentional, but—but nevertheless, it kept happening. So like one of the reasons it happened was this stupid thing where they switched pharmacies, and the f—the shape and the size of the pill changed, so they thought it was, you know, a different level than it was.. - cuz it matched the other—you know, like silly things like that... understand, like you have the routine. You—your nor—you—you’d know what it looks like. You’d grab it, you know, but anyway, so that’s why she kept being in so much pain, and that was, you know, excruciating, obviously, to watch.(2)  And he was not really diagnosed 'til, um, November, and he, you know—with the proper diagnosis. So his—he, uh, was getting all kinds of medicine and stuff, you know, and he wasn't—you know, I—it was probably making him worse. (4)  when they actually were giving him the wrong medication, and I said those five days of him really literally in a comatose—I thought that's when he was dying— they brought hospice in. Um, once we adjust—took him off that medication, he came back just fine, but hospice stayed with us that entire time. Um, he did have to get, um, re—I don't know if recertify is the right word—- every three months. (18)  when he went to that facility, I said to them, "Watch what—you know, we need to be careful." They wouldn't—they didn't—as, obviously, didn't call. Um, a couple of months ago, we had an incident where they—that medication was still in his chart, and one of the caregivers actually didn't pay attention and go further into it. And he was agitated, and she gave that to him. And I, of course, came in—and they have—they have to call you an—with any—it's by law they call the—call me to say, "Well, we gave him the wrong medication." And, sure enough, he slept for 36 hours. (18)  **Trying medications that weren’t helping**  other than after those—all those falls. Then we couldn’t do that because she was in too much pain. Um, she just couldn’t stand the pain. It was so painful she couldn’t even be lured to go shopping (2)  She was on morphine, and she was on an anti-anxiety. And I finally figured out that it was the, um, anti-anxiety—and they had her on—I think it was Ativan, at first, and it wasn't working as well. (3)  it's, like, just give 'em morphine, and we keep 'em comfortable. But, um, I think sometimes—and I think I've read this—that, you know, with Lewy body, they don't always react to medications the same way. And-and I actually think the morphine was probably h-helping to make her feel more anxious, and it just—you know, I don't think that that was the right medication for her. But you know, they-they did get it changed. (3)  everything on Lewy body is to, quote, “regulate their meds.” If-if I heard that once, I’ve heard it a hundred times.And it’s like they—it’s a cocktail, and they have to get it just right. Well, in her case, it never got just right (6)  And Dad had not done well on any of the medications they had tried…. But they were not successful in finding a drug that would help him. (9)  Well, they prepared us very clearly that the hope was we could get medications for him that would help him, but it might not work. (9)  So then—so then they put him on Ativan, and Lorazepam, and that, uh, uh, that he was on until he, uh—well, after the-the-the vomiting part, then it got more we would, uh, crush it, put it in the side of his mouth. In a little water, you know. And then it got, uh, where that wasn’t doing any good (30)  **Ending Medications**  'Cause at that point—I mean, he had had all these medicines, and they took him off the medications he had been on in the hospital. And I think he was just on morphine at that point. (4)  We had to make the decision to stop, uh, the carbidopa-levodopa. Yeah, because of the side effects it was causing. My mom clinically went insane— at the end (8)  So, we stopped the carbidopa-levodopa and the Aricept on the 16^th^ because when the hospice doctor came out, uh, my mom was hi—super confused. She had not experienced that kind of confusion before, and just—uh, it was distressing to me because there—she just wasn’t—and that’s when, you know, the doctor was very blunt—and I appreciated that—um, that my mom was—you know, had-had lost her mind. She was insane, and-and so, she was dis—in distress (8)  we stopped the carbidopa-levodopa and the, um, the, uh, Aricept… She was insane, and-and so, she was dis—in distress, even though she wasn’t verbally able to communicate that anymore…we had to make the decision of continuing on with the medication that was causing, you know, uh, quite a bit of this, or stopping and knowing that it was gonna cause a catastrophic failure—- uh, physically for her. And that’s what we chose to do. (8)  Nothing-nothing even flowed together correctly. And that was within a week of stopping Namenda. So, when I asked, they were like, “No, no, it’s not the medication.” But, you know, I may—I’m wrong, I don’t have an M.D., but—I may be wrong… (8)  once we start the carbid—uh, stopped the carbidopa-levodopa, she lost all muscle. (8)  I think they took him off his medication. I had ‘em stop it. Um, like about four months before he passed. Because he couldn’t swallow anymore. Yes, he was—it was very hard for him to swallow, so we made the decision of getting him off of everything and He seemed to be okay, but you know, we knew it was—it was getting to the end. (11)  then it got to the point where, you know, she—I guess, s-skipping right to the very end, she wasn’t—started—w-not being able to take her meds, which they hadup for her, and so that was hard. So then I-I just said, you know, “No more meds. There’s no really point to have any—having any meds anymore.” Um, so maybe a little—it would be helpful to have a little bit more information as to when it’s okay to do that, I think, because I feel like it—if-if you’re a caregiver, you may f—you may feel that stopping her medication means you’ve given up on her. And it doesn’t necessarily mean that. It just means that you’re actually kind of lessening a bit of anxiety— for her, because she can’t swallow it. She can’t swallow it, and if it’s too hard, it’s too hard. cuz she had transitioned to—gosh, she was on—she was on mushy food for—I-I can’t remember; quite a—quite a while. (14)  Um, you know, he was way o—he’d been off meds for two weeks. He’d been off insulin for two weeks, obviously. No intake, so why bother? (23)  So, when he, {Husband}, was released from the hospital, um, I called Dr. {Name}, and he came to (Name} Village, and we met in the library. Um, my s-son, my stepson, had come from {Washington State}, and, uh, my stepson, was on, um, a conference call. And we met with him. And it was that trust, and he explained to me why it would not be advisable to keep him on, um, th-the antibiotics, which— actually, the doctor gave me the antibiotic, um, um, prescription just because I-I was in shock. I said, “No, no. You can’t stop it now.” [Laughter] You know? ….Uh, and everybody had been—knew him in the nurse—uh, in the memory care which was on the same campus, but in a different—and that was also one of the reasons that I brought—took him off and put him in hospice, because, um, I trusted Dr. {Name}. (26)  **Patient ended Medications**  Dr.{Name} does want you to take this medication. I want you to take the medication, but I can’t make you take it. But it’s right here when you’re ready to take it.” And sometimes she would. Most times she wouldn’t…. “I know, but she’s been so good. And there’s no way to prove it, but I—” and I told him, I said, “I-I would swear in a court of law that the galantamine worked for her.” And, um, when she stopped taking it, um, and when I couldn’t get her to take it, it was actually just sh—when she wasn’t taking it at all, um, is really when I started noticing the decline. (7) |

| Resources regarding expectations for end of life | **Online**  I think being as—having as much information as possible about what to expect—I-I did a little research on the Lewybodydementia.org website and found—- a whole bunch of blog postings, I think from the UK, I think, given the way that they were spelling words and whatnot. And-and apparently, people with Lewy body dementia do end up starving to death. Um, and that’s-that—knowing that ahead of time, like knowing that in April, might have helped me clue in. Um, not everybody will starve to death, but it-it-it—there seem to be a number of them who said, “Yeah, my loved one—uh we f—we fixed his favorite or her favorite meal, and she just wouldn’t eat. She just had no appetite, and the next thing you know is, you know, there y—there you go.” (23)  **Hospice written education**  the hospice people had given us, you know, like a nice, you know, reader on like-There’s this stage and then that stage, and then this stage and then that stage. (2)  a pamphlet about hospice and medications, or—I’m not exactly sure, cuz I—would I have been in the right fr—mindset to-to make the—make right decisions? But I feel like maybe if I-I would have—or uh maybe my brother, who was kind of doing more of the boy stuff, right, it seemed like more of the black-and-white stuff, whereas I was—- more involved in the emotion [chuckles], you know? Maybe he would have been able to assist with that, but I just di—I feel like we had a little lack of information as far as meds and how it goes. they are—you know, as the days progress, we’re assuming that her kidneys are shut down and they’re gonna hurt her, so we’re give her more morphine, or—yeah, how does that—how does that go dow—go down? I think that would be helpful. (14)  they gave us all this literature and—-we were kinda like going by what and that morning.. (19)  Um, I did a lotta research on the Internet. There’s a lotta great information on hospice, so kudos-kudos to hospice for having lots and lots of great information. (23)  at the end um, she—again, hospice guided us with um, um, uh cuz we had we-we-we had been taking her bed out to be in the sunshine and uh, in front of the TV where shows made her laugh and uh, or, you know smile.Uh, but then uh, it became, you know, you’re—they gave us a fantastic handbook. Oh god, it was so good. And we saw what the book said and what they were saying was that her world—she started preparing to pass. You know, even with dementia and withdrawing. And so, she—we kept her in her-her bedroom, which was huge and sunny. Um, we did um, the baths. We uh, uh, sat by her side. (24)  But um, that little booklet really helped us, because I looked for profusion. I looked, you know and um, so um, uh, because, I think, we had such a great hospice group, we had that little book that helped, you know, my sister tremendously. My brother was an MD before he took over the company, so he knew what was going on. But uh, me, you know, it helped us and the care givers. They had Spanish version, because down here in Texas it’s Spanish. And um—- it helped us know to be ready. (24)  **Education from Facebook group(s)**  I I found this Facebook group really helpful. Um, I didn’t mention that. I thought that was really useful. I joined an LBD Facebook group. It’s like a private group, and, um, I didn’t post a lot, but just seeing how—it’s, uh, seeing the story happening in so many different places in similar ways, and some different ways. But just that—that group of people was useful, and that could be done more formally, I think. (2)  I got the most information from Facebook groups of other people who were taking care of Lewy body— That's where I got most of my information, and the best information, not from any of the medical providers, at all. (3)  …on Facebook, there are so many—some of ‘em are private, obviously, but, um, even in just, uh, you know, they—Caregivers of Dementia is one of the groups in there. And people are hungry for the information. (8)  -But it is different. It’s a different animal, and— and you’re right. There’s little to nothing out there, really. And, um, it’s frustrating. (7)  And my life saver, um, was an online lewy body support group on Facebook. And where you could share with other caregivers. You know, you’re noticing this or what has worked for constipation or, um, I guess early on, uh, uh, the falls and, and the postural issues. (13)  {Name}—he’s the one Facebook. Um, he had done video tapes with his mom. Um, he’s brought kind of a public face to living, you know, in a changing condition of lewy body dementia and, um, the more education that we can do, um, you know, the better. (13)  This was another thing I wish that I had known about when I needed it. And so I—I'm always—I'm also on a Facebook caregiver group, and I'm always recommending that, again, so you ‑ you know kind of what's coming and, uh, it doesn't shock you, and you don't think—you know, there are all these issues like tube feeding and ‑ and medications, and should you give this or that to prolong life? Um, and I talk to my people in the Facebook group about, you know, are you doing this for him, or are you doing it for you? (15)  Now we're in the day of age where all of 'em were online and Googling and whatever you do to find out on their own level of the knowledge that they wanted to know about Lewy body dementia and—and whatever they knew about end of life. The daughter that was here—she was with me a lot, and so she actually—so she would somewhat talk with them a little bit about it also. (18)  **Journals**  I sort of have access to a lot of the medical literature. You know, I could find no journal—I found a few journal articles that kinda spoke to sorta the average lifespan of a living patient being about three years- after an initial diagnosis. But um, I don’t know how accurate that was. That was just an article. I just—there was such a uh, uh, dearth of information that uh, it was—it was really challenging…(10)  **Books**  I would want everyone to read *Being Mortal (2)*  …there’s one Lewy body book, I think, that I bought as soon as she was diagnosed. Do you know that book, by a husband and wife couple? I bought it. I bought it for a couple people. Yeah, it was really good. It’s the only one I found to m—like care a—Lewy body caregiving something. It’s a husband and wife. Uh, there it is. A Caregiver’s Guide to Lewy Body—it was 2010. And it was um by the Whitworths… Caregiver’s Guide to Lewy Body Dementia, and it was actually a really, really good book. I bought several and shared them. Well, it seemed to be more since then. Okay, that’s good, but that was back when I was in the—in the midst of it, 2010 when that was the only book. Um, but maybe even some sort of little, you know, information. (14)  it's a ‑ a book also by a hospice nurse. I'm always forgetting her name. Nancy something. It's called "Gone from My Sight". And that, to me, was extremely helpful. (15)  when you first get diagnosed there’s a book out there, and I got it, and I tell you the one thing I didn’t do is I didn’t read it to my husband and I wish I would have. It’s called “Life in the Balance.” It’s doctor, it was written by an MD out of Boston. He was a cardiologist, well known. And he had Parkinson’s with lewy body. And he wrote it before he got so bad. And he talks about how hard it is to deal with this disease in the early stages and what he was afraid of. It’s a wonderful book, Life in the Balance. And I think anybody that is diagnosed with this should get this book. And the caregiver should read it. It doesn’t, it, it’s not end of life kinds of things, but it’s about I’ve been diagnosed with this, I’m a cardiologist, what do I do now. Because - what I’ve found is most people that I met with lewy body they’re really smart people. [Chuckling] And they know how to work around what they know is going on in their body. Like my husband fooled us for a long time. I think he had the disease for quite a while before I recognized—I knew something was wrong, but I didn’t know what. And, and so this book when I read it, it was like an ah ha moment for me. And I’m almost willing to give this book out to anybody that’s diagnosed. I would be willing to do that, uh, as a contribution because it, it just, I read it, a part of it last night again. Uh, I hadn’t read it for two years, you know three years. And I wish I would have had it earlier than later. (21)  I bought some books by people that, on lewy body that, you know, written by a caregiver and that, but I will say that Life in the Balance, um, and this doctor was still alive when he wrote the book. It’s I think 2008. I think he’s passed away now. But, you know, because he was a doctor he knew how important it was to share what was going on. I bought some books by people that, on lewy body that, you know, written by a caregiver and that, but I will say that Life in the Balance, um, and this doctor was still alive when he wrote the book. It’s I think 2008. I think he’s passed away now. But, you know, because he was a doctor he knew how important it was to share what was going on. I have to lay in bed and actually force myself into my body before I can get out of bed. (21)  it was a fabulous book that I gave to everybody and stole from and everything. *The 36-Hour Day*. (24)  **Youtube**  discovered in my research this Youtube, and apparently, it's a ‑ a book also by a hospice nurse. I'm always forgetting her name. Nancy something. It's called "Gone from My Sight". (15)  **Other**  I still have the CD here. Um, I gave them both general printed materials and, um, a DVD, uh, about caring for people with Lewy body—- um, by Teepa Snow…. Uh, she’s an occupational therapist who works with patients with various forms of dementia, but she has produced materials specifically about, um, Lewy body. And, uh, she’s a very—um, she does a lot of acting-out scenarios, and-and—- she’s both entertaining and informative. So, yeah, I really recommend her. (20*) Speaking of information given to the memory care unit* |
| --- | --- |

| Religious experiences | **Religious aspect to hospice not helpful for those who aren’t religious**  I had a heck of a time finding-findi—well, I always ask the questions, uh, uh, question, are, you know, do-do you serve atheists? And they always serve all faiths. Well, that does not mean— no faith. Cuz we had no faith. We had no faith, and we fought it all the time. Um, e-even when I chose non-religious places, um, we confronted that. And h-he was a jerk about it. Even when he didn’t know—have his mind. (5)  I was referred to, for like caregivin-caregiving counseling. And, um, one o—one of my griefs was just religious affiliations and-and, um, I-I mean, I felt like I was getting my hand patted and sent away. Um, so, there—I mean, there’s plenty of spiritual guidance, but when you’re not spiritual, it’s not guidance, you know?... I was a real bitch about it. (5)  He wouldn’t say that he was an atheist, but he was agnostic, and he—disliked, um, um, being prayed over, and he thought that was hokey. Um, and I-I made an effort to protect him for that—from that. And, for insta—for instance, when he—when he first became ill, he picked up the phone and he called the local hospice to come out. And he didn’t—I don’t think he perceived that he was calling the Catholic hospital for that. (5)  they sent a nun out, you know, to speak to him. And, um, I knew that would make him angry, you know? So, then they sent another person out. I’m like, “Well, another person isn’t good,” you know, just because it’s, um, um, when you have the—when you have those feelings, those, uh, end-of-life, um, death-with-dignity feelings, you don’t pick up the phone and call a Catholic hospital. (5)  well, I always ask the questions, uh, uh, question, are, you know, do-do you serve atheists? And they always serve all faiths. Well, that does not mean— no faith. Cuz we had no faith. We had no faith, and we fought it all the time. Um, e-even when I chose non-religious places, um, we confronted that. And h-he was a jerk about it. Even when he didn’t know—have his mind (5)  The hospice people were not warm and fuzzy, but they were very—um, you know, they did the right things. There was a chaplain who kept coming in and sort of standing over me, creeping me out, and so I told—no, really, it was-it-it made me feel very uncomfortable. First of all we-we’re not religious, but it made me feel that the private time just to be with him, even though he wasn’t that aware—but he was aware. It’s like—um, so I did speak with one of the other hospice people and say I need the chaplain not to come in here. He didn’t. (22)  **Religious aspects to hospice helpful**  She said, “Well, you know, Donna, if you don’t want hospice for any other reason, think about this. If you have hospice, they can pronounce her when she dies. You don’t have to wait. And, you know, for the medical examiner to come or all-all that. And I said, “You know, I never—I never realized that.” (7)  They provide a priest, uh and, you know, you don’t have to be religious. (6)  we're Catholic and so we, um, had the priest come and give him the last rights. And, um, also he was able to take a tiny bit of the host, which made Mom feel really good and we- all of us—- feel really good. (9)  We had a chaplain that came in every other week (18)  . And I think one of the ministers, the priest came and he said to me he said, “This is the time when your husband, the person,” he said, “I’ve been in a hospital setting for a long time, and that when this stage comes the person feels scared. And you, the best thing you could do is be there for them, hold their hand, talk to them and give them the assurance that he, that they need.” And those were one of the most *[voice cracking 09:04]* comforting words I’ve ever heard. And I’ve never heard that from a pastor or—but this was a priest that I had come in and give my husband the last right. (21)  the last night, um, the Hospice minister came and he said, “Can I come until like 6:00?” I said, “Oh I don’t wanna bother you.” And he said, “I think this is the night {Wife’s name}. I just have a feeling. Now the Hospice nurse would not tell me that. But he came and he told my husband, and it was just amazing, he said, “{Husband’s name} you’ve worked,”—he was a physicist [voice cracking]— and he worked in fiber optics. And he said “{Husband} you have worked in light all your life. Go to the light.” And my husband died about four hours later. (21)  The-the best thing that happened with {Name of Palliative Care Company} is we’re Jewish, and - um, a-a Jewish, uh, {Name}, who is the chaplain for the Jewish people, came around, uh, early on, when he had first got out of the hospital. She came with a bag fuporum, and I had a chance to talk to her and explain what was happening. And she was really, um—she-she was there for us, um, at the end. Um, I called her in the middle of the night. {Name} stayed with me, and I thought, “I’ve gotta—I don’t know what prayers to say.” And I asked to call her, and-and the nurse said, “Well, we can’t call until 7:00 in the morning.” We called, and, um, {Name} is her name. And {Name} said, “Well, um, it will take me about an hour and a half to get there. Is that okay?” And I said, “Certainly.” And she arrived, um, 10 minutes after he had passed. We were still—- at the bedside, my son and I. And, uh, she was really, um, just a wealth of-of assistance. Um, just full of comfort, you would say— um, at that point. Um, and, uh, she stayed...(26)  I met, the-the chaplain. In fact, she and I have hit a friendship, and, um, she’s coming over later this week. Uh, we’re both pianists, so [laughs] that kinda you know, enabled that (28) |
| --- | --- |
